# Effects of ultrashort laser pulses on angular distributions of photoionization spectra

**DOI:** 10.1038/s41598-017-05915-8

**Published:** 2017-07-27

**Authors:** C. H. Raymond Ooi, W. L. Ho, A. D. Bandrauk

**Affiliations:** 1 Department of Physics, Faculty of Science, University of Malaya, 50603 Kuala Lumpur, Malaysia; 2 Laboratoire de Chimie Théorique, Faculté des Sciences, Université de Sherbrooke, Sherbrooke, Québec, J1K 2R1 Canada

## Abstract

We study the photoelectron spectra by intense laser pulses with arbitrary time dependence and phase within the Keldysh framework. An efficient semianalytical approach using analytical transition matrix elements for hydrogenic atoms in any initial state enables efficient and accurate computation of the photoionization probability at any observation point without saddle point approximation, providing comprehensive three dimensional photoelectron angular distribution for linear and elliptical polarizations, that reveal the intricate features and provide insights on the photoionization characteristics such as angular dispersions, shift and splitting of photoelectron peaks from the tunneling or above threshold ionization(ATI) regime to non-adiabatic(intermediate) and multiphoton ionization(MPI) regimes. This facilitates the study of the effects of various laser pulse parameters on the photoelectron spectra and their angular distributions. The photoelectron peaks occur at multiples of 2*ħω* for linear polarization while  odd-ordered peaks are suppressed in the direction perpendicular to the electric field. Short pulses create splitting and angular dispersion where the peaks are strongly correlated to the angles. For MPI and elliptical polarization with shorter pulse﻿s﻿ the peaks split into doublets and the first peak vanishes. The carrier envelope phase(CEP) significantly affects the ATI spectra while the Stark effect shifts the spectra of intermediate regime to higher energies due to interference.

## Introduction

Intense light-matter interaction has been extensively studied in recent years, particularly strong field photoionization^1^ and ultrafast^2, 3^ laser physics. A laser pulse with peak intensity $\frac{1}{2}c{\varepsilon }_{0}{|{E}_{0}|}^{2}$ (1 a.u. = 3.5 × 10^16^ W/cm^2^; 1 a.u. for energy *I*_0_ = 27.2 eV, frequency $\omega =4.2\times {10}^{16}\,{s}^{-1}$, wavelength *λ* = 45 nm) or equivalently the electric field strength of *E*_0_ = 5 × 10^9^ Vcm^−1^ can now be easily achieved, providing experimental tools covering a wide range of phenomena from perturbative nonlinear optics of multiphoton ionization (MPI)^4^ to nonperturbative processes, above threshold ionization (ATI), high harmonic generation (HHG)^5–9^, nonsequential double ionization (NSDI), etc. Development of theoretical techniques of intense light-matter interactions^10, 11^ and experimental studies of ultrafast electron dynamics in the nonperturbative regime have led to applications in imaging of ultrafast processes^12^ in atoms and molecules using photoelectrons angular distribution, particularly probing ultrafast molecular dynamics through laser-induced electron diffraction (LIED)^13^. Recollision of electrons in both linear and bichromatic circular polarizations^14^ is used for molecular imaging^15, 16^.

*Keldysh*: When the ponderomotive energy ${U}_{p}=\frac{{e}^{2}{E}^{2}}{4{m}_{e}{\omega }^{2}}$ for electric field strength *E* and frequency *ω* is lower than the ionization potential *I*_*p*_, the multiphoton ionization is dominant as a perturbative process where *n*-number of photons are absorbed as the electron makes a transition from the ground state to the continuum, with the Keldysh parameter $\gamma =\frac{\omega \sqrt{2{m}_{e}{I}_{p}}}{eE}=\sqrt{\frac{{I}_{p}}{2{U}_{p}}}\gg 1$. When *U*_*p*_ is in the order of or larger than *I*_*p*_ tunnelling ionization^17^ process occurs where an electron escapes from the distorted potential barrier under the influence of the intense laser field, with $\gamma \ll 1$, i.e., low *I*_*p*_, low frequency *ω* and large electric field *E*. The Keldysh formalism^18^ provides correct qualitative descriptions not only for the two limiting cases, but also the intermediate case where $\gamma \simeq 1$ ^19^, covering a broad range of frequencies^20^. The theory is even more powerful than generally believed as it yields accurate quantitative results for negative ions with short-range potentials^21^. The Keldysh theory has been extended further by others^22–24^ into the PPT theory^25^, the ADK theory^26^, and the KFR theory to higher order perturbative terms by Faisal^27^ and Reiss^28^. Tunnelling ionization theory was extended^29^ to study the influence of relativistic effects on photoelectrons in arbitrary initial states on the angular distribution of electrons^30^. Recently the original Keldysh theory that was valid the quadratic photoelectron momenta ${p}^{2}/\sqrt{2{m}_{e}{I}_{p}}$ has been generalized to be valid for arbitrary momenta^31^.

*TDSE*: Piraux^32^ provided one of the earliest theoretical descriptions that is in qualitative agreement with experimental data^33^ for femtosecond pulse photoionization of a highly excited hydrogen atom using time-dependent Schrodinger equation (TDSE) and Floquet theory. In HHG, full numerical approach was used to obtain the harmonic spectra of atoms, ions^34^ and molecules^35^. However, for photoionization^36^, there are not many theoretical treatments^37^ that include the temporal effects of arbitrary laser pulses into the Keldysh-type formalism for a wide range of laser field strength *E*, frequency *ω*, and duration *t*_*p*_, especially in the non-adiabatic (intermediate) regime where rapid pulse turn-on and off^38^ dominates as in recent experimental work involving excitations by attosecond pulses^39^. A partial Fourier-transform approach to tunnel ionization^40^ can recover all analytical results for ATI with arbitrary bound potential but only for static field.

*Motivation*: Ultrashort (approaching the attosecond time scale), circularly and elliptically-polarized pulses are leading to a new science (ultrafast and strong field physics)^41^ that requires generalisation of previous strong field models and theories beyond SFA and saddle point methods. Martiny and Madsen studied the effects of ellipticity (or helicity) on photoelectron momentum distribution using the Keldysh theory, both with and without the saddle point method^42^, and the effects of CEP using TDSE^43^. However, the effects of ultrashort laser pulse and the CEP on the angular distribution of the photoelectron spectra for different regimes of Keldysh parameter and laser polarization (for linear and circular) have not been systematically studied. Besides, in the case without saddle point method the Keldysh theory involves cumbersome three-fold numerical integrations of the matrix element. Development of semi-analytical approach that avoids the three-fold numerical integration for the study would be useful and relevant to current development of high field physics with ultrashort pulses, especially, the emphasis on polarization effects, which are being investigated in the generation of attosecond pulse^44, 45^ and bright table-top X-ray pulses^46^.

*Objective*: Our main objective here is to study the effects of short pulse excitations, polarization and Stark shifts on the angular dependence of photoelectron spectra beyond the Keldysh-saddle point approach. In this paper, we generalize the Keldysh formalism by developing a computationally efficient semi-analytical approach for hydrogenic atom in arbitrary bound state interacting with an intense laser pulse of arbitrary time-dependence, duration *t*_*p*_, field polarization and carrier envelope phase (CEP) *φ*, valid for tunnelling and intermediate, and to some extent the multiphoton regimes, *without* using the stationary phase or the saddle point approximation.

**Figure 1:**
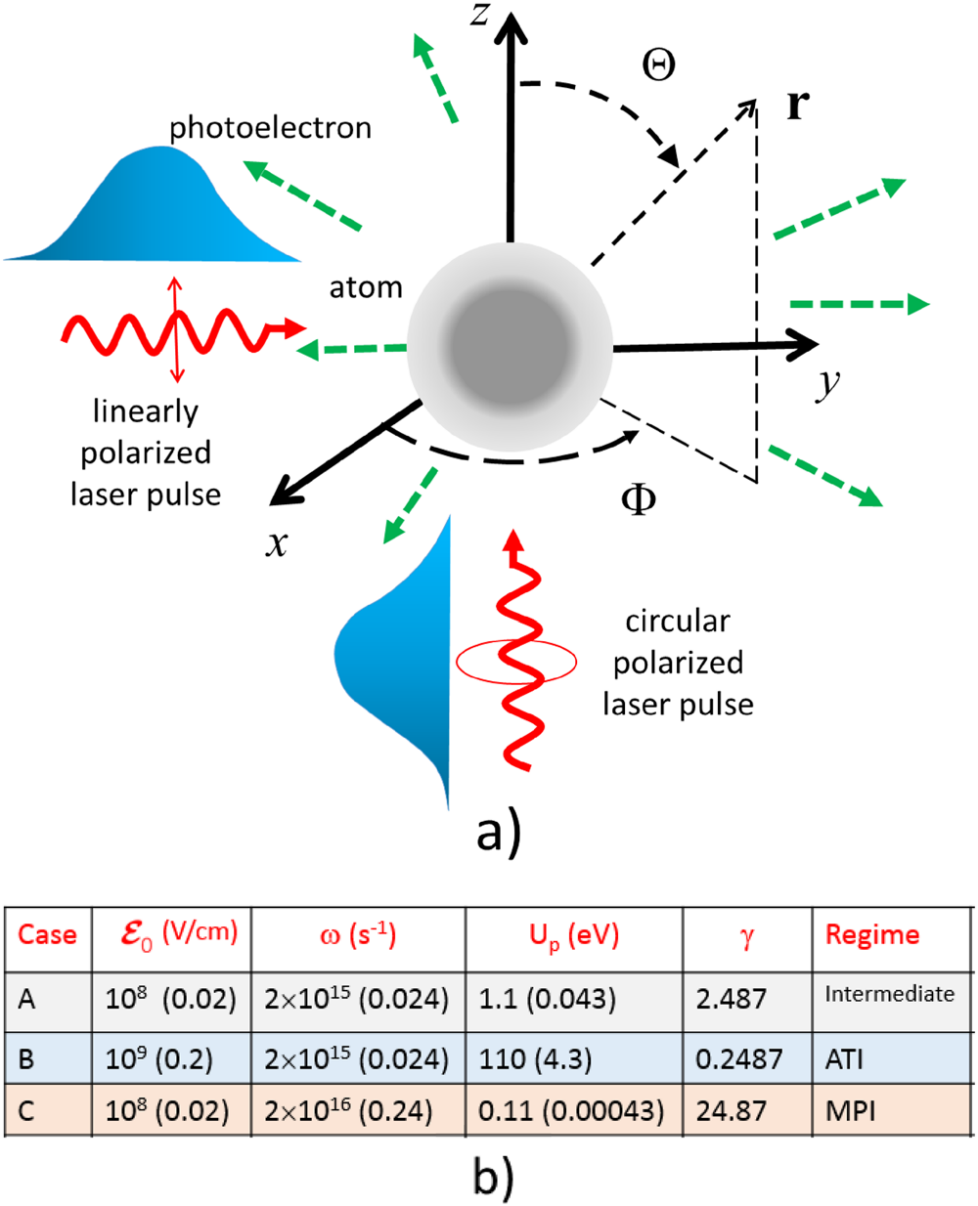
(**a**) Illustration showing the directions of the linearly polarized and circularly polarized laser pulses with respect to a spherical coordinate system. The directionality of the photoelectron is defined by the angles Θ and Φ. The numbers in the brackets are in atomic units (a.u.). (**b**) Table showing the 3 cases characterized by Keldysh parameter *γ* for laser field amplitude ${ {\mathcal E} }_{0}$ and frequency *ω* : A (intermediate regime), B (ATI regime), C (MPI regime). The case of ${ {\mathcal E} }_{0}={10}^{9}\,{{\rm{Vcm}}}^{-1}$ and $\omega ={10}^{16}\,{{\rm{s}}}^{-1}$ has the same *γ* as case A and therefore gives the same spectral distributions as case A except with higher probabilities.

*Approach*: The ac Stark shift has been shown to give important effects in strong field interactions^47, 48^. Recently it was shown^49^ that off-resonant modulation of dynamical Stark shifts can produce extremely short laser pulses. Previous treatments neglected laser-induced Stark shifts^30, 31^ but it is now included here. The stationary phase approximation and further analytical treatment on saddle point are not possible when finite pulse duration and Stark effects are included. However, using a parabolic function instead of the Gaussian envelope to simulate the laser pulse and incorporating the Fourier transform of the initial hydrogenic (bound) wavefunctions to obtain analytical transition matrix element (bypassing the 3D spatial integration), we are able to obtain semi-analytical approach that facilitates efficient, rapid and accurate computation of the 3D angular distribution of photoelectron spectra compared to full numerical evaluation of the Keldysh theory without the saddle point approximation. This approach is in between the full TDSE and the analytical Keldysh model. It allows us to analyze the effects of pulse length, direction of observation and laser polarization (illustrated in Fig. 1).

*Validity*: As long as *γ* is not too small or too large the Keldysh method is valid. The original Keldysh approximation is applicable over a wide range of frequencies^20^ under the condition $F={E}_{0}/{E}_{C}\ll 1$ where ${E}_{C}\approx 5\times {10}^{11}\,V/\,{\rm{m}}$ is the Coulomb electric field at Bohr radius. We do not include the Coulomb interaction to the Volkov state^50^ as it only introduces a prefactor^51^ to the ionization rate, and therefore, alters the overall order of magnitude but does not significantly affect the many qualitative physical results. For excited states and elliptical polarization^52^ the Coulomb field will be less important than the electric field.

## Linear and quadratic Stark shift effects

We formulate the model for pulse excitations of general atomic initial (bound) states taking into account the ac Stark effect within the strong field approximation^53^. We assume that the atomic initial state eigenvalue *I*_*nlm*_(*t*) varies with time parametrically or adiabatically according to the dynamical first order and second order Stark shift terms

1 $${I}_{nlm}(t)=-\frac{{I}_{p}}{{n}^{2}}+{a}_{nlm}E(t)+{b}_{nlm}E{(t)}^{2},$$

where *n*, *l*, *m* are the principle quantum number, azimuthal quantum number and also magnetic quantum number respectively. |*I*_*nlm*_(*t*)| corresponds to the ionization energy that varies with the energy level, *n*, *l*, *m* in the presence of time varying electric field (ac Stark effect) and ${I}_{p}={I}_{100}=\frac{{m}_{e}}{2{\hslash }^{2}}{(\frac{{e}^{2}}{4\pi {\varepsilon }_{0}})}^{2}=13.6\,{\rm{eV}}$, for the 1 s state of the hydrogen atom.

The coefficients *a*_*nlm*_ and *b*_*nlm*_ are coefficients for the linear and quadratic Stark shifts, 2*b*_*nlm*_ is also referred to, as quasistatic dynamic polarizability. Specific details of *b*_*nlm*_ for atomic Cs can be found in Eqs 5 and 6 of ref. 54, but it is not necessary for our present study as it is sufficient to estimate the order of magnitude $\frac{{\Vert d\Vert }^{2}}{\hslash \delta }$ where ||*d*|| (~10^−30^ Cm) is reduced dipole matrix element and *δ* (~5 × 10^13^ s^−1^) is the laser detuning from typical atomic transitions. For field magnitude of 10^11^ V/m, the quadratic Stark shift is in the order^55^ of *I*_*p*_.

The Stark shifted eigenenergy satisfies the TDSE ${I}_{nlm}(t){\rm{\Xi }}(t)=\frac{1}{i\hslash }\frac{\partial }{\partial t}{\rm{\Xi }}(t)$ as part of the initial bound state wavefunction

2 $${\psi }_{nlm}({\bf{r}},t)\simeq {\psi }_{nlm}({\bf{r}})\,{\rm{\Xi }}(t)$$

3 $${\rm{\Xi }}(t)=\exp \,(-\frac{i}{\hslash }{\int }_{0}^{t}{I}_{nlm}(t^{\prime} )dt^{\prime} ),$$

which is similar to Eq. 12 of ref. 47 while ${\psi }_{nlm}({\bf{r}})$ is assumed to be the undistorted adiabatic initial wavefunction that satisfies the time independent Schrödinger equation of the bound system with time independent unperturbed eigenvalue $\frac{{I}_{p}}{{n}^{2}}$

4 $$\frac{{I}_{p}}{{n}^{2}}{\psi }_{nlm}({\bf{r}})=[-\frac{{\hslash }^{2}{\nabla }^{2}}{2{m}_{e}}+V({\bf{r}})]\,{\psi }_{nlm}({\bf{r}}){\rm{.}}$$

for electron binding potential energy $V({\bf{r}})\sim \frac{{e}^{2}}{4\pi {\varepsilon }_{0}{a}_{0}}=27.2\,{\rm{eV}}$ much greater than the dipole interaction with the laser field $V({\bf{r}})\gg e{a}_{0}{ {\mathcal E} }_{0}$ (or ${ {\mathcal E} }_{0}\ll 5\times {10}^{11}\,\,{\rm{V}}/{\rm{m}}$) where *a*_0_ is the Bohr radius and ${ {\mathcal E} }_{0}$ is the electric field amplitude when the electron is mainly bound to the nucleus.

In other words, we assume ${V}_{L}({\bf{r}},t)=e{\bf{E}}(t)\cdot {\bf{r}}$ to be much smaller than $V({\bf{r}})$ when the electron is still strongly attached to the nucleus at the onset of ionization with the corresponding wavefunction ${\psi }_{nlm}({\bf{r}})$. The perturbative Stark-shifted correction energy to the eigenvalue *I*_*nlm*_ is ${a}_{nlm}E(t)+{b}_{nlm}E{(t)}^{2}$ depends parametrically on time through the time dependent electric field **E**(*t*). The adiabatic approximation of Eq. 2 has been shown to yield qualitatively agreeable results with exact numerical results^56^ as the time variation due to the laser pulse affects the phase through *I*_*nlm*_(*t*) more sensitively than the transition matrix element through the wavefunction ${\psi }_{nlm}({\bf{r}})$.

Once the electron is ionized from the nucleus, the potential energy falls off rapidly and the spatiotemporal dynamics of the electron with quasi-momentum **p** is well represented by the Volkov wavefunction

5 $${\psi }_{{\bf{p}}}^{V}({\bf{r}},t)=\frac{1}{\sqrt{\mathcal{V}}}\,\exp \{\frac{i}{\hslash }{\rm{\Pi }}(t)\cdot {\bf{r}}\}\,\exp \{-\frac{i}{\hslash }[\frac{1}{2{m}_{e}}{\int }_{0}^{t}{\rm{\Pi }}{(\tau )}^{2}d\tau ]\}$$

where ${\rm{\Pi }}(t)={\bf{p}}-q{\bf{A}}(s)$ is the generalized momentum and *q* = −*e*, as the solution of the TDSE for ionized electron

6 $$i\hslash \frac{\partial }{\partial t}{\psi }_{{\bf{p}}}^{V}({\bf{r}},t)=[-\frac{{\hslash }^{2}{\nabla }^{2}}{2{m}_{e}}+{V}_{L}({\bf{r}},t)]\,{\psi }_{{\bf{p}}}^{V}({\bf{r}},t){\rm{.}}$$

## Keldysh Formalism for General Pulse-Shape

We start with the original Keldysh formalism to study the effects of pulse duration and observation direction on the photoionization spectra. The general wavefunction is in a superposition of discrete initial bound states and continuum free electron Volkov states, ${\rm{\Psi }}({\bf{r}},t)={\sum }_{nlm}\,{a}_{nlm}(t){\psi }_{nlm}({\bf{r}},t)+{\sum }_{{\bf{p}}}\,c({\bf{p}},t){\psi }_{{\bf{p}}}^{V}({\bf{r}},t)$. To focus on the pulse and the Stark effects we may neglect the free-free transitions and the bound-bound transitions, which is essentially the Keldysh formalism. A pulse with 10 fs duration has a bandwidth corresponding to 0.07 eV much smaller than the transition energy between the ground and the first excited state. Thus, the probability amplitude for the photoelectron momentum **p** simplifies to involve only a single bound initial state ${{\boldsymbol{\psi }}}_{s}({\bf{r}})$ with the free electron continuum,

7 $$c({\bf{p}},s)\simeq \frac{1}{i\hslash {\omega }_{{\rm{0}}}}{\int }_{0}^{s}\,{V}_{0}(s^{\prime} )\,\exp \{i\mathcal{S}(s^{\prime} )\}ds^{\prime} ,$$

with the transition matrix element (subscript “0” indicating without Coulomb correction)

8 $${V}_{0}(s^{\prime} )=-\int \,{\psi }_{s}({\bf{r}}){V}_{L}{\psi }_{{\bf{p}}}^{V}({\bf{r}},s^{\prime} ){d}^{3}r=e\int \,{\psi }_{s}({\bf{r}})\,{\bf{E}}(s^{\prime} )\cdot {\bf{r}}\,\exp (-\frac{i}{\hslash }{\rm{\Pi }}(s^{\prime} )\cdot {\bf{r}})\frac{{d}^{3}r}{\sqrt{\mathcal{V}}},$$

that can be evaluated analytically for hydrogenic bound state ${\psi }_{s}({\bf{r}})$ and the important action phase

9 $$\mathcal{S}(s^{\prime} )=\frac{1}{\hslash {\omega }_{0}}{\int }_{0}^{s^{\prime} }[-{I}_{nlm}(s^{\prime\prime} )+K(s^{\prime\prime} )]ds^{\prime\prime} ,$$

where $K=\frac{{{\rm{\Pi }}}^{2}}{2{m}_{e}}$ is the kinetic energy and $\mathcal{V}$ is the normalization volume.

The roles of the laser source will be elaborated in the following section where we introduce the pulse envelope function, *g*(*t*) that characterizes the time-dependent electric field of the laser pulse (with amplitude ${ {\mathcal E} }_{0}$) for linear polarization **E**_*lin*_ or elliptical polarization **E**_*elp*_,

10 $$(\begin{array}{c}{{\bf{E}}}_{lin}\\ {{\bf{E}}}_{elp}\end{array})={ {\mathcal E} }_{0}f^{\prime} (t)\,\hat{n}(t)={ {\mathcal E} }_{0}g(t)\,\hat{n}(t\mathrm{).}$$

Here $\hat{n}$ is the harmonic time-dependent polarization vector with absolute carrier-envelope phase (CEP) $\phi$ defined by

11 $$\hat{n}(s)=(\begin{array}{c}\hat{z}\,\cos \,(\frac{\omega }{{\omega }_{0}}s+\phi )\\ \hat{x}\alpha \,\cos \,(\frac{\omega }{{\omega }_{0}}s+\phi )+\hat{y}\beta \,\sin \,(\frac{\omega }{{\omega }_{0}}s+\phi )=\frac{1}{2}(\hat{x}\alpha -i\hat{y}\beta ){e}^{i(\frac{\omega }{{\omega }_{0}}s+\phi )}+\frac{1}{2}(\hat{x}\alpha +i\hat{y}\beta ){e}^{-i(\frac{\omega }{{\omega }_{0}}s+\phi )}\end{array}),$$

where we have introduced the dimensionless time *s* = *ω*_0_ *t* with *ω*_0_ chosen such that *ω*_0_ × pulse duration 1261.

The vector potential, **A** satisfies ${\bf{E}}=-\frac{\partial {\bf{A}}}{\partial t}$ and can be obtained by performing integration by parts,

12 $$(\begin{array}{c}{{\bf{A}}}_{lin}(t)\\ {{\bf{A}}}_{elp}(t)\end{array})=-\frac{1}{{\omega }_{0}}{\int }_{-\infty }^{t}{\bf{E}}(t)\,{\omega }_{0}dt=-\frac{{ {\mathcal E} }_{0}}{{\omega }_{0}}\hat{a}(s),$$

where the general normalized vector potential is

13 $$\hat{a}(s)={\int }_{-\infty }^{s}g(s)\,\hat{n}(s)ds=(\begin{array}{c}\hat{z}{{\rm{\Sigma }}}_{c}\\ \hat{x}\alpha {{\rm{\Sigma }}}_{c}+\hat{y}\beta {{\rm{\Sigma }}}_{s}\end{array})$$

14 $$=\,{f(s)\,\hat{n}(s)|}_{-\infty }^{s}-{\int }_{-\infty }^{s}f(s^{\prime} )\,\hat{n}^{\prime} (s^{\prime} )\,ds^{\prime} \,{\rm{or}}$$

15 $$=\,{g(s)\,\hat{m}(s)|}_{-\infty }^{s}-{\int }_{-\infty }^{s}g^{\prime} (s^{\prime} )\,\hat{m}(s^{\prime} )\,ds^{\prime} ,$$

16 $$\hat{a}^{\prime} (s)=\frac{d\hat{a}(s)}{ds}=g(s)\,\hat{n}(s)=g(s)\,\hat{m}^{\prime} (s)=f^{\prime} (s)\,\hat{n}(s),$$

where $$\hat{m}(s)=\int \hat{n}(s)ds$$.

For a c.w. laser, the pulse envelope function is a constant, *g* = 1, *g*′ = 0 and the vector potential reduces to that in our previous work^31^. Note that there are 2 possible expressions for $\hat{a}(s)$ (above) by partial integration and one still has to go through the evaluation of the (integral) second term in $\hat{a}$ after partial integration. Thus, only the second version is useful for sufficiently slow varying envelopes, where the electric field envelope (not the vector potential) vanishes asymptotically $g(s\to -\infty )=0$ and *g*′ is sufficiently small that the second term involving the integral of $\hat{a}$ may be neglected, hence

$$\hat{a}(s)\simeq {g(s)\hat{m}(s)|}_{-\infty }^{s}=g(s)(\begin{array}{c}\hat{z}\,\sin \,(\tfrac{\omega }{{\omega }_{0}}s+\phi )\\ \hat{x}\alpha \,\sin \,(\tfrac{\omega }{{\omega }_{0}}s+\phi )-\hat{y}\beta \,\cos \,(\tfrac{\omega }{{\omega }_{0}}s+\phi )\end{array}).$$

For certain pulse shape, such as Gaussian and Lorentzian functions it is possible to evaluate $\hat{a}(s)$ exactly without the need for the slowly varying envelope approximation (SVEA). However, these functions turn out to have complex arguments. We find that parabolic envelope function leads to analytical results that are simpler as shown in the Appendix A and will be used to simulate the pulse shapes.

## Action Phase for General Pulses

For electric field strength exceeding 10^12^ Vcm^−1^, the electron is beyond the classical limit and relativistic correction needs to be included in the TDSE, particularly generalization of the kinetic energy term^30^, ${K}_{rel}={m}_{e}{c}^{2}(\sqrt{1+{\xi }^{2}}-1)$ where $\xi (s)=\frac{{\rm{\Pi }}(s)}{{m}_{e}c}$. Here, we restrict to the non-relativistic case and the action phase can be written as (we drop subscript *nlm* to simplify notations) so that it is possible to obtain an analytical expression from

17 $$\mathcal{S}(s)=\frac{1}{\hslash {\omega }_{0}}{\int }_{0}^{s}\,[-I+\frac{{\rm{\Pi }}{(s)}^{2}}{2{m}_{e}}]\,ds$$

18 $$\begin{array}{c}=\,\frac{1}{\hslash {\omega }_{0}}\{(\frac{{I}_{p}}{{n}^{2}}+K)\,s+\frac{q{{\mathcal{E}}}_{0}}{{m}_{e}{\omega }_{0}}{\int }_{0}^{s}{\bf{p}}\cdot \hat{a}ds^{\prime} +2{U}_{p0}{\int }_{0}^{s}\hat{a}\cdot \hat{a}ds^{\prime} -{a}_{nlm}{{\mathcal{E}}}_{0}{\int }_{0}^{s}\,g|\hat{n}|ds^{\prime} \\ -{b}_{nlm}{{\mathcal{E}}}_{0}^{2}{\int }_{0}^{s}{g}^{2}\hat{n}\cdot \hat{n}ds^{\prime} \}\end{array}$$

where $${U}_{p0}=\frac{{e}^{2}{ {\mathcal E} }_{0}^{2}}{4{m}_{e}{\omega }_{0}^{2}}$$ and $$K=\frac{{p}^{2}}{2{m}_{e}}$$.

The integrals in Eq. 18 to be evaluated can be expressed as

19 $$\begin{array}{c}{\int }_{0}^{s}g|\hat{n}|ds^{\prime} =\,{\int }_{0}^{s}g(s^{\prime} )(\begin{array}{c}\cos \,(\frac{\omega }{{\omega }_{0}}s^{\prime} +\phi )\\ \sqrt{{\alpha }^{2}\,{\cos }^{2}\,(\frac{\omega }{{\omega }_{0}}s^{\prime} +\phi )+{\beta }^{2}\,{\sin }^{2}\,(\frac{\omega }{{\omega }_{0}}s^{\prime} +\phi )}\end{array})\,ds^{\prime} \\ \quad \quad \quad \quad \,\,\simeq \,(\begin{array}{c}{{\rm{\Delta }}{\rm{\Sigma }}}_{c}\\ \sqrt{{\alpha }^{2}{{\rm{\Delta }}{\rm{\Sigma }}}_{c}^{2}+{\beta }^{2}{{\rm{\Delta }}{\rm{\Sigma }}}_{s}^{2}}\end{array}),\end{array}$$

20 $$\begin{array}{c}{\int }_{0}^{s}{g}^{2}\hat{n}\cdot \hat{n}ds^{\prime} =\,{\int }_{0}^{s}g{(s^{\prime} )}^{2}(\begin{array}{c}{\cos }^{2}\,(\frac{\omega }{{\omega }_{0}}s^{\prime} +\phi )\\ {\alpha }^{2}\,{\cos }^{2}\,(\frac{\omega }{{\omega }_{0}}s^{\prime} +\phi )+{\beta }^{2}\,{\sin }^{2}\,(\frac{\omega }{{\omega }_{0}}s^{\prime} +\phi )\end{array})\,ds^{\prime} \\ \quad \quad \quad \quad \quad \quad =\,(\begin{array}{c}{{\rm{\Delta }}{\rm{\Sigma }}}_{c}^{\mathrm{(2)}}\\ {\alpha }^{2}{{\rm{\Delta }}{\rm{\Sigma }}}_{c}^{\mathrm{(2)}}+{\beta }^{2}{{\rm{\Delta }}{\rm{\Sigma }}}_{s}^{\mathrm{(2)}}\end{array}),\end{array}$$

21 $${\int }_{0}^{s}{\bf{p}}\cdot \hat{a}ds^{\prime} ={\int }_{0}^{s}(\begin{array}{c}{p}_{z}{{\rm{\Sigma }}}_{c}\\ {p}_{x}\alpha {{\rm{\Sigma }}}_{c}+{p}_{y}\beta {{\rm{\Sigma }}}_{s}\end{array})\,ds^{\prime} =(\begin{array}{c}{p}_{z}{{\rm{\Xi }}}_{c}\\ {p}_{x}\alpha {{\rm{\Xi }}}_{c}+{p}_{y}\beta {{\rm{\Xi }}}_{s}\end{array}),$$

22 $${\int }_{0}^{s}\hat{a}\cdot \hat{a}ds^{\prime} ={\int }_{0}^{s}(\begin{array}{c}{{\rm{\Sigma }}}_{c}^{2}\\ {\alpha }^{2}{{\rm{\Sigma }}}_{c}^{2}+{\beta }^{2}{{\rm{\Sigma }}}_{s}^{2}\end{array})\,ds^{\prime} =(\begin{array}{c}{{\rm{\Xi }}}_{c}^{\mathrm{(2)}}\\ {\alpha }^{2}{{\rm{\Xi }}}_{c}^{\mathrm{(2)}}+{\beta }^{2}{{\rm{\Xi }}}_{s}^{\mathrm{(2)}}\end{array}),$$

where ${{\rm{\Sigma }}}_{s,c}(s)$, ${{\rm{\Sigma }}}_{s,c}^{\mathrm{(2)}}(s)$, ${{\rm{\Xi }}}_{s,c}(s)$ and ${{\rm{\Xi }}}_{s,c}^{\mathrm{(2)}}(s)$ in the above integrals are given in Appendix A and ${\rm{\Delta }}{\rm{\Sigma }}={\rm{\Sigma }}(s)-{\rm{\Sigma }}\mathrm{(0)}$, with the upper(lower) element for linear (elliptical) polarization.

Note that for elliptical polarization we face the difficulty to analytically integrate Eq. 19, due to the first order or linear Stark shifts. The linear Stark term is negligible for centrosymmetric systems such as atoms and symmetric molecules, contributes only for degenerate levels such as *n* = 2 of hydrogen atom by mixing the degenerate states equally, eg, 2*s* ± 2*p* for “static” fields only. Besides, for time dependent fields there is no first order Stark shift. Only the second order time dependent Stark shift remains which can be important for strong fields in the case of pulsed lasers, especially for excited states as it is comparable to the ponderomotive energies for Rydberg states, as found in ref. 57.

The action phase can be written as

23 $$\begin{array}{c}{{\rm{\Omega }}}_{0}\mathcal{S}=(\frac{1}{{n}^{2}}+{\wp }^{2})s-2{\lambda }_{0}(\begin{array}{c}{\wp }_{z}{{\rm{\Xi }}}_{c}\\ {\wp }_{x}\alpha {{\rm{\Xi }}}_{c}+{\wp }_{y}\beta {{\rm{\Xi }}}_{s}\end{array})+{\lambda }_{0}^{2}(\begin{array}{c}{{\rm{\Xi }}}_{c}^{\mathrm{(2)}}\\ {\alpha }^{2}{{\rm{\Xi }}}_{c}^{\mathrm{(2)}}+{\beta }^{2}{{\rm{\Xi }}}_{s}^{\mathrm{(2)}}\end{array})\\ \quad \quad \,\,\,\,\,-\,{\bar{a}}_{nlm\mathrm{,0}}{\lambda }_{0}(\begin{array}{c}{{\rm{\Sigma }}}_{c}\\ \sqrt{{\alpha }^{2}{{\rm{\Sigma }}}_{c}^{2}+{\beta }^{2}{{\rm{\Sigma }}}_{s}^{2}}\end{array})-{\bar{b}}_{nlm\mathrm{,0}}{\lambda }_{0}^{2}(\begin{array}{c}{{\rm{\Sigma }}}_{c}^{\mathrm{(2)}}\\ {\alpha }^{2}{{\rm{\Sigma }}}_{c}^{\mathrm{(2)}}+{\beta }^{2}{{\rm{\Sigma }}}_{s}^{\mathrm{(2)}}\end{array}),\end{array}$$

in terms of the dimensionless parameters (that is helpful for numerical computation)

24 $${{\rm{\Omega }}}_{0}=\frac{\hslash {\omega }_{0}}{{I}_{p}};\,\vec{\wp }=\frac{{\bf{p}}}{\sqrt{2{m}_{e}{I}_{p}}};\,{\lambda }_{0}=\frac{\omega }{{\omega }_{0}}/\gamma =\frac{\omega }{{\omega }_{0}}\frac{e{ {\mathcal E} }_{0}}{\omega \sqrt{2{m}_{e}{I}_{p}}},$$

25 $${\bar{a}}_{nlm\mathrm{,0}}={a}_{nlm}\frac{{\omega }_{0}}{e}\sqrt{\frac{2{m}_{e}}{{I}_{p}}};\,{\bar{b}}_{nlm\mathrm{,0}}={b}_{nlm}\frac{2{m}_{e}{\omega }_{0}^{2}}{{e}^{2}},$$

26 $${\bar{a}}_{nlm\mathrm{,0}}{\lambda }_{0}={{\rm{\Omega }}}_{0}{a}_{nlm}{ {\mathcal E} }_{0}\frac{1}{\hslash {\omega }_{0}}={a}_{nlm}\frac{{ {\mathcal E} }_{0}}{{I}_{p}}$$

27 $${\bar{b}}_{nlm\mathrm{,0}}{\lambda }_{0}^{2}={b}_{nlm}\frac{{ {\mathcal E} }_{0}^{2}}{{I}_{p}}$$

with the Keldysh parameter $\gamma =\sqrt{\frac{{I}_{p}}{2{U}_{p}}}$ and ${U}_{p}=\frac{{e}^{2}{E}^{2}}{4{m}_{e}{\omega }^{2}}$. According to Eq. 23 the coefficient of the quadratic term ${ {\mathcal E} }_{0}^{2}$ can be zero when $\hat{a}\cdot \hat{a}\simeq {\bar{b}}_{nlm\mathrm{,0}}{g}^{2}\hat{n}\cdot \hat{n}$.

## Transition Matrix Element for Arbitrary Pulses

The transition of the electron of hydrogen atom from an arbitrary energy level, ${\psi }_{nlm}$ to Volkov state, ${{\rm{\Psi }}}_{p}({\bf{r}},t)$ under the interaction of laser pulse, can be described by computing the transition matrix element using the hydrogenic wavefunction for arbitrary initial state as

28 $${V}_{0}(s)=e{ {\mathcal E} }_{0}g(s)\int \,{\psi }_{s}({\bf{r}})\hat{n}(s)\cdot {\bf{r}}\,\exp (-\frac{i}{\hslash }{\rm{\Pi }}(s)\cdot {\bf{r}})\frac{{d}^{3}r}{\sqrt{\mathcal{V}}}{\rm{.}}$$

Performing the volume integration numerically would be very time consuming. We find that the transition matrix element can be evaluated analytically by noting that the integral $\int {\psi }_{s}({\bf{r}})\hat{n}(s)\cdot {\bf{r}}\,\exp \,(-\tfrac{1}{\hslash }{\rm{\Pi }}(s)\cdot {\bf{r}})\frac{{d}^{3}r}{\sqrt{\mathcal{V}}}$ is actually a Fourier transform and has an analytical expression

29 $$\begin{array}{rcl}\frac{\sqrt{{\mathrm{(2}\pi \hslash )}^{3}}}{\sqrt{\mathcal{V}}}i\hslash \hat{n}\cdot {\nabla }_{{\rm{\Pi }}}{\bar{\psi }}_{s}({\rm{\Pi }}) & = & \frac{1}{\sqrt{V{Y}^{3}}}i\hslash \hat{n}\cdot {\nabla }_{{\rm{\Pi }}}\{FGH\}\end{array}$$

30 $$\begin{array}{rcl} & = & \frac{1}{Y\hslash }i\hslash \hat{n}\cdot {\nabla }_{\zeta }\{FGH\},\end{array}$$

where ${\bar{\psi }}_{s}({\rm{\Pi }})=F({\rm{\Phi }}^{\prime} )G({\rm{\Theta }}^{\prime} )H({\rm{\Pi }})$ is the Fourier transform of the initial wavefunction. *VY* ^3^ = 1 and the dimensionless quantities are defined as $\zeta =\frac{{\rm{\Pi }}}{Y\hslash }=\frac{n{\rm{\Pi }}}{Z\sqrt{2{m}_{e}{I}_{p}}}=\frac{n{a}_{0}{\rm{\Pi }}}{Z\hslash }=\frac{n}{Z}p^{\prime}$, $Y=Z/n{a}_{0}=Z\eta /n$, $\eta =\sqrt{2{m}_{e}{I}_{p}}/\hslash =1/{a}_{0}$, $p^{\prime} ={\rm{\Pi }}/\sqrt{2{m}_{e}{I}_{p}}$. The expressions for *F*, *G* and *H* have been worked out by Podolsky and Pauling^58^ for general hydrogenic state as

31 $$F({\rm{\Phi }}^{\prime} )=\frac{1}{{(2\pi )}^{\mathrm{1/2}}}{e}^{\pm im{\rm{\Phi }}^{\prime} }$$

32 $$G({\rm{\Theta }}^{\prime} )={(\frac{(2l+1)(l-m)!}{2(l+m)!})}^{\frac{1}{2}}{P}_{l}^{m}(\cos \,{\rm{\Theta }}^{\prime} )$$

33 $$H(\zeta )=-{(-i)}^{l}\pi {2}^{2l+4}l!{(\frac{n(n-l-1)!}{(n+l)!})}^{\frac{1}{2}}\frac{{\zeta }^{l}}{{({\zeta }^{2}+1)}^{l+2}}{C}_{n-l-1}^{l+1}(\frac{{\zeta }^{2}-1}{{\zeta }^{2}+1})$$

where ${P}_{l}^{m}$ are the associated Legendre polynomials and ${C}_{n-l-1}^{l+1}$ are the Gegenbauer polynomials. The explicit definitions for ${\rm{\Pi }}$, ${\rm{\Theta }}^{\prime}$, ${\rm{\Phi }}^{\prime}$ are given in the Appendix B.

Therefore, the transition matrix *V*_0_(*s*) can be expressed as

34 $${V}_{0}(s)=e{ {\mathcal E} }_{0}g(s)\frac{i}{Y}\hat{n}\cdot {\nabla }_{\zeta }\{FGH\},$$

clearly showing the pulse-shape dependence with the above dot product evaluated analytically as

35 $$\begin{array}{c}M(s)=\hat{n}(s)\cdot {\nabla }_{\zeta }\{FGH\}\\ \quad \quad \,=\,(\begin{array}{c}\cos (\tfrac{\omega }{{\omega }_{0}}s+\phi )\,(\cos {\rm{\Theta }}^{\prime} \tfrac{\partial H}{\partial \zeta }FG-\,\sin {\rm{\Theta }}^{\prime} \tfrac{1}{\zeta }\tfrac{\partial G}{\partial {\rm{\Theta }}^{\prime} }FH)\\ \alpha \cos (\tfrac{\omega }{{\omega }_{0}}s+\phi )\,(\sin {\rm{\Theta }}^{\prime} \cos {\rm{\Phi }}^{\prime} \tfrac{\partial H}{\partial \zeta }FG+\,\cos {\rm{\Theta }}^{\prime} \cos {\rm{\Phi }}^{\prime} \tfrac{1}{\zeta }\tfrac{\partial G}{\partial {\rm{\Theta }}^{\prime} }FH-\tfrac{\sin {\rm{\Phi }}^{\prime} }{\zeta \sin {\rm{\Theta }}^{\prime} }\tfrac{\partial F}{\partial {\rm{\Phi }}^{\prime} }GH)\\ +\,\beta \sin (\tfrac{\omega }{{\omega }_{0}}s+\phi )\,(\sin {\rm{\Theta }}^{\prime} \sin {\rm{\Phi }}^{\prime} \tfrac{\partial H}{\partial \zeta }FG+\,\cos {\rm{\Theta }}^{\prime} \sin {\rm{\Phi }}^{\prime} \tfrac{1}{\zeta }\tfrac{\partial G}{\partial {\rm{\Theta }}^{\prime} }FH+\tfrac{\cos {\rm{\Phi }}^{\prime} }{\zeta \sin {\rm{\Theta }}^{\prime} }\tfrac{\partial F}{\partial {\rm{\Phi }}^{\prime} }GH)\end{array}){\rm{.}}\end{array}$$

The required derivatives $\frac{\partial F({\rm{\Phi }}^{\prime} )}{\partial {\rm{\Phi }}^{\prime} }$, $\frac{\partial G({\rm{\Theta }}^{\prime} )}{\partial {\rm{\Theta }}^{\prime} }$ and $\frac{\partial H(\zeta )}{\partial \zeta }$ are given in the Appendix C. If we adopt the Coulomb-Volkov wavefunction^50^ where the Volkov plane wave has to be multiplied by the normalized continuum state of hydrogen that depends on **r** through the hypergeometric function, thus it would be complicated to obtain analytical matrix element.

Finally, the photoionization amplitude can be evaluated numerically as

36 $$c({\bf{p}},s)=\frac{e{ {\mathcal E} }_{0}}{Y\hslash {\omega }_{{\rm{0}}}}{\int }_{0}^{s}\,g(s^{\prime} )M(s^{\prime} )\,\exp \{i\mathcal{S}(s^{\prime} )\}\,ds^{\prime} $$

Equations 18, 35 and 36 together are semi-analytical expressions that provide convenient computation of the transient photoionization probability density $P({\bf{p}},s)={|{c}_{b}({\bf{p}},s)|}^{2}$ of atoms in any excited^59^ initial state by laser pulses with arbitrary shape, width, polarization and CEP.

Other computable quantities involving photoionization are the transient photoionization rate of a particular momentum $\frac{dP({\bf{p}},s)}{ds}=2{\lambda }_{0}^{2}{\rm{Re}}\,[{c}_{b}({\bf{p}},s){M}^{\ast }(s)\,\exp \,\{-i\frac{{\rm{\Phi }}(s)}{{{\rm{\Omega }}}_{0}}\}]$ and the total photoionization rate $w(s)=\frac{V}{{\mathrm{(2}\pi \hslash )}^{3}}$ ${\int }_{-\infty }^{\infty }\,\frac{dP({\bf{p}},s)}{dt}{d}^{3}p$. Using ${d}^{3}p={p}^{2}dpd{{\rm{\Omega }}}_{a}$ the rate over the solid angle ${{\rm{\Omega }}}_{a}$ can be obtained as $\frac{dw}{d{{\rm{\Omega }}}_{a}}\,=\,$ $\frac{\mathcal{V}}{{\mathrm{(2}\pi \hslash )}^{3}}{\int }_{0}^{\infty }\,\frac{dP({\bf{p}})}{dt}{p}^{2}dp.$

## Analysis of Stationary Phase Approximation

We next explore the saddle point approximation with the possibility of using the approximate expression ${\int }_{C}\,g(z){e}^{sf(z)}dz\to \sqrt{\frac{2\pi }{sf^{\prime\prime} ({z}_{0})}}g({z}_{0}){e}^{sf({z}_{0})}$ to find the semi-analytical expression for the photoionization probability in the steady state

37 $$P({\bf{p}})=2\pi {\lambda }_{0}^{2}\,{|\sum _{s}\frac{M({s}_{s})}{\sqrt{{\mathcal{S}}^{^{\prime\prime} }({s}_{s},q)}}\exp \{i\mathcal{S}({s}_{s},q)\}|}^{2},$$

which requires evaluation of the second order derivative of the phase using $\hat{a}^{\prime} (s)=g(s)\hat{n}(s)$,

38 $$\begin{array}{c}{{\rm{\Omega }}}_{0}\mathcal{S}^{\prime\prime} =-2{\lambda }_{0}\vec{\wp }\cdot \hat{a}^{\prime} +{\rm{2}}{\lambda }_{0}^{2}\hat{a}^{\prime} \cdot \hat{a}-{\bar{a}}_{nlm\mathrm{,0}}{\lambda }_{0}\{g^{\prime} |\hat{n}|+g|\hat{n}|^{\prime} \}\\ \,\,\quad \,-\,2{\bar{b}}_{nlm\mathrm{,0}}{\lambda }_{0}^{2}\hat{a}^{\prime} \cdot (g^{\prime} \hat{n}+g\hat{n}^{\prime} )\end{array}$$

while the first order derivative is

39 $${{\rm{\Omega }}}_{0}\frac{d\mathcal{S}}{ds}=(\frac{1}{{n}^{2}}+{\wp }^{2})-2{\lambda }_{0}\overrightarrow{\wp }\cdot \hat{a}-{\bar{a}}_{nlm\mathrm{,0}}{\lambda }_{0}g|\hat{n}|-{\bar{b}}_{nlm\mathrm{,0}}{\lambda }_{0}^{2}{g}^{2}\hat{n}\cdot \hat{n}+{\lambda }_{0}^{2}\hat{a}\cdot \hat{a},$$

40 $$\begin{array}{c}=\,(\frac{1}{{n}^{2}}+{\wp }^{2})-2{\lambda }_{0}(\begin{array}{c}{\wp }_{z}{{\rm{\Sigma }}}_{c}\\ {\wp }_{x}\alpha {{\rm{\Sigma }}}_{c}+{\wp }_{y}\beta {{\rm{\Sigma }}}_{s}\end{array})+{\lambda }_{0}^{2}(\begin{array}{c}{{\rm{\Sigma }}}_{c}^{2}\\ {\alpha }^{2}{{\rm{\Sigma }}}_{c}^{2}+{\beta }^{2}{{\rm{\Sigma }}}_{s}^{2}\end{array})\\ \quad -\,{\bar{a}}_{nlm\mathrm{,0}}\,{\lambda }_{0}g(s)(\begin{array}{c}\cos \,(\frac{\omega }{{\omega }_{0}}s+\phi )\\ {n}_{c}(s)\end{array})-{\bar{b}}_{nlm\mathrm{,0}}{\lambda }_{0}^{2}{g}^{2}(s)(\begin{array}{c}{\cos }^{2}\,(\frac{\omega }{{\omega }_{0}}s+\phi )\\ {n}_{c}^{2}(s)\end{array}),\end{array}$$

where

41 $${n}_{c}=\sqrt{{\alpha }^{2}\,{\cos }^{2}\,(\frac{\omega }{{\omega }_{0}}s+\phi )+{\beta }^{2}\,{\sin }^{2}\,(\frac{\omega }{{\omega }_{0}}s+\phi )}$$

with the corresponding unit vectors, and their magnitudes

42 $$\hat{n}=(\begin{array}{c}\hat{z}\,\cos \,(\frac{\omega }{{\omega }_{0}}s+\phi )\\ \hat{x}\alpha \,\cos \,(\frac{\omega }{{\omega }_{0}}s+\phi )+\hat{y}\beta \,\sin \,(\frac{\omega }{{\omega }_{0}}s+\phi )\end{array}),$$

43 $$\hat{n}^{\prime} =(\begin{array}{c}-\hat{z}\,\sin \,(\frac{\omega }{{\omega }_{0}}s+\phi )\\ -\hat{x}\alpha \,\sin \,(\frac{\omega }{{\omega }_{0}}s+\phi )+\hat{y}\beta \,\cos \,(\frac{\omega }{{\omega }_{0}}s+\phi )\end{array}),$$

44 $$|\hat{n}|=(\begin{array}{c}\cos \,(\frac{\omega }{{\omega }_{0}}s+\phi )\\ \sqrt{{\alpha }^{2}\,{\cos }^{2}\,(\frac{\omega }{{\omega }_{0}}s+\phi )+{\beta }^{2}\,{\sin }^{2}\,(\frac{\omega }{{\omega }_{0}}s+\phi )}\end{array}),$$

45 $$|\hat{n}|^{\prime} =(\begin{array}{c}-\sin \,(\frac{\omega }{{\omega }_{0}}s+\phi )\\ \frac{-({\alpha }^{2}-{\beta }^{2})\,\sin \,2(\frac{\omega }{{\omega }_{0}}s+\phi )}{\sqrt{2}\sqrt{{\alpha }^{2}\,{\cos }^{2}\,(\frac{\omega }{{\omega }_{0}}s+\phi )+{\beta }^{2}\,{\sin }^{2}}}\end{array}){\rm{.}}$$

Equation 39 does not lead to an analytical solution for the stationary points (subscript’s’) in time when $\mathcal{S}^{\prime} =0$ unless the Stark terms are neglected. For linear polarization we have $(\frac{1}{{n}^{2}}+{\wp }^{2})-2{\lambda }_{0}{\wp }_{z}{{\rm{\Sigma }}}_{c}+{\lambda }_{0}^{2}{{\rm{\Sigma }}}_{c}^{2}=0$,

46 $${{\rm{\Sigma }}}_{c}({s}_{s})=\frac{1}{{\lambda }_{0}}({\wp }_{z}\pm i\sqrt{\frac{1}{{n}^{2}}+{\wp }_{\perp }^{2}}),$$

where ${\wp }_{\perp }=\sqrt{{\wp }_{x}^{2}+{\wp }_{y}^{2}}$ is the transverse normalized momentum, giving the analytical probability

47 $$P({\bf{p}})=2\pi {\lambda }_{0}^{2}{{\rm{\Omega }}}_{0}{|\sum _{s}\frac{M({s}_{s})\exp [\frac{i}{{\Omega }_{0}}\{(\frac{1}{{n}^{2}}+{\wp }^{2}){s}_{s}-2{\lambda }_{0}{\wp }_{z}{{\rm{\Xi }}}_{c}({s}_{s})+{\lambda }_{0}^{2}{{\rm{\Xi }}}_{c}^{\mathrm{(2)}}({s}_{s})\}]}{\sqrt{2{\lambda }_{0}{\wp }_{z}g({s}_{s})\cos (\tfrac{\omega }{{\omega }_{0}}{s}_{s}+\phi )}}|}^{2}.$$

## Reduction to Results of CW

In the continuous wave (cw) limit we set the pulse envelope *g*(*s*) → 1, so

48 $$\hat{a}(s)\to {\int }_{-\infty }^{s}\,(\begin{array}{c}\hat{z}\,\cos \,(\frac{\omega }{{\omega }_{0}}s+\phi )\\ \hat{x}\alpha \,\cos \,(\frac{\omega }{{\omega }_{0}}s+\phi )+\hat{y}\beta \,\sin \,(\frac{\omega }{{\omega }_{0}}s+\phi )\end{array})\,ds=\frac{{\omega }_{0}}{\omega }(\begin{array}{c}\hat{z}S\\ \hat{x}\alpha S-\hat{y}\beta C\end{array}),$$

where $C(s)=\cos \,(\frac{\omega }{{\omega }_{0}}s+\phi )$, $S(s)=\sin \,(\frac{\omega }{{\omega }_{0}}s+\phi )$. For cw case, the derivative of the action phase simplifies to

49 $$\begin{array}{c}{{\rm{\Omega }}}_{0}\mathcal{S}^{\prime} =\frac{1}{{n}^{2}}+{\wp }^{2}-2\lambda (\begin{array}{c}{\wp }_{z}S\\ {\wp }_{x}\alpha S-{\wp }_{y}\beta C\end{array})+{\lambda }^{2}(\begin{array}{c}{S}^{2}\\ {\alpha }^{2}{S}^{2}+{\beta }^{2}{C}^{2}\end{array})\\ \quad \quad \,-\,{\bar{a}}_{nlm}\,\lambda (\begin{array}{c}C\\ \sqrt{{\alpha }^{2}{C}^{2}+{\beta }^{2}{S}^{2}}\end{array})-{\bar{b}}_{nlm}{\lambda }^{2}(\begin{array}{c}{C}^{2}\\ {\alpha }^{2}{C}^{2}+{\beta }^{2}{S}^{2}\end{array})\end{array}$$

where $$\lambda ={\lambda }_{0}(\frac{{\omega }_{0}}{\omega })=\frac{e{ {\mathcal E} }_{0}}{\omega \sqrt{2{m}_{e}{I}_{p}}}$$ and $${\bar{a}}_{nlm\mathrm{,0}}\,{\lambda }_{0}={\bar{a}}_{nlm}\,\lambda $$, $${\bar{a}}_{nlm\mathrm{,0}}={\bar{a}}_{nlm}(\frac{{\omega }_{0}}{\omega })$$.

Using ${{\rm{\Sigma }}}_{c}^{\mathrm{(2)}}\to \int {C}^{2}ds^{\prime} =\frac{1}{2}[s+\frac{{\omega }_{0}}{\omega }SC]$, ${{\rm{\Sigma }}}_{s}^{\mathrm{(2)}}\to \int {S}^{2}ds^{\prime} =\frac{1}{2}[s-\frac{{\omega }_{0}}{\omega }SC]$ with ${{\rm{\Xi }}}_{c}=\int {{\rm{\Sigma }}}_{c}ds^{\prime} =-{(\frac{{\omega }_{0}}{\omega })}^{2}C$, ${{\rm{\Xi }}}_{s}\,=$ $\int {{\rm{\Sigma }}}_{s}ds^{\prime} =-{(\frac{{\omega }_{0}}{\omega })}^{2}S$, $\,{{\rm{\Xi }}}_{c}^{\mathrm{(2)}}={(\frac{{\omega }_{0}}{\omega })}^{2}\int {S}^{2}ds^{\prime} ={(\frac{{\omega }_{0}}{\omega })}^{2}\frac{1}{2}[s-\frac{{\omega }_{0}}{\omega }SC]$, $\,{{\rm{\Xi }}}_{s}^{\mathrm{(2)}}={(\frac{{\omega }_{0}}{\omega })}^{2}\int {C}^{2}ds^{\prime} ={(\frac{{\omega }_{0}}{\omega })}^{2}\frac{1}{2}[s+\frac{{\omega }_{0}}{\omega }SC]$ the phase becomes

50 $$\begin{array}{c}{{\rm{\Omega }}}_{0}\mathcal{S}=(\frac{1}{{n}^{2}}+{\wp }^{2})s+2\lambda (\begin{array}{c}\frac{{\omega }_{0}}{\omega }{\rm{\Delta }}C{\wp }_{z}\\ \frac{{\omega }_{0}}{\omega }{\rm{\Delta }}C{\wp }_{x}\alpha +\frac{{\omega }_{0}}{\omega }{\rm{\Delta }}S{\wp }_{y}\beta \end{array})\\ \quad \quad \quad +\,{\lambda }^{2}(\begin{array}{c}\frac{1}{2}[s-\frac{{\omega }_{0}}{\omega }{\rm{\Delta }}(SC)]\\ {\alpha }^{2}\frac{1}{2}[s-\frac{{\omega }_{0}}{\omega }{\rm{\Delta }}SC]+{\beta }^{2}\frac{1}{2}[s+\frac{{\omega }_{0}}{\omega }{\rm{\Delta }}(SC)]\end{array})\\ \quad \quad \quad -\,{\bar{a}}_{nlm}\lambda (\begin{array}{c}(\frac{{\omega }_{0}}{\omega }){\rm{\Delta }}S\\ (\frac{{\omega }_{0}}{\omega })\sqrt{{(\alpha {\rm{\Delta }}S)}^{2}+{(\beta {\rm{\Delta }}C)}^{2}}\end{array})\\ \quad \quad \quad -\,{\bar{b}}_{nlm}{\lambda }^{2}(\begin{array}{c}\frac{1}{2}[s+\frac{{\omega }_{0}}{\omega }{\rm{\Delta }}(SC)]\\ {\alpha }^{2}\frac{1}{2}[s+\frac{{\omega }_{0}}{\omega }{\rm{\Delta }}(SC)]+{\beta }^{2}\frac{1}{2}[s-\frac{{\omega }_{0}}{\omega }{\rm{\Delta }}(SC)]\end{array}),\end{array}$$

For linear polarization one obtains

51 $${{\rm{\Omega }}}_{0}\mathcal{S}^{\prime\prime} (s,\vec{\wp })=-2\lambda C[{\wp }_{z}-\lambda \mathrm{(1}+{\bar{b}}_{nlm})S]+{\bar{a}}_{nlm}\,\lambda S,$$

52 $${{\rm{\Omega }}}_{0}\mathcal{S}=(\frac{1}{{n}^{2}}+{\wp }^{2})\,s+\lambda (\frac{{\omega }_{0}}{\omega })\,[2{\wp }_{z}{\rm{\Delta }}C(s)-{\bar{a}}_{nlm}{\rm{\Delta }}S(s)]+{\lambda }^{2}\frac{1}{2}{\rm{\Delta }}R(s),$$

where ${\rm{\Delta }}x(s)=x(s)-x\mathrm{(0)}$, $x=C,S,R$ functions are defined as $C(s)=\cos \,(\frac{\omega }{{\omega }_{0}}s+\phi )$, $S(s)=\sin \,(\frac{\omega }{{\omega }_{0}}s+\phi )$ and $R(s)=\mathrm{(1}-{\bar{b}}_{nlm})s-\frac{1}{2}\frac{{\omega }_{0}}{\omega }\mathrm{(1}+{\bar{b}}_{nlm})\,\sin \,2(\frac{\omega }{{\omega }_{0}}s+\phi )$.

If the linear Stark is neglected ${\bar{a}}_{nlm}=0$ for linear polarization, the stationary phase approximation gives $\frac{1}{{n}^{2}}+{\wp }^{2}-2\lambda {\wp }_{z}S+{\lambda }^{2}{S}^{2}-{\bar{b}}_{nlm}{\lambda }^{2}\mathrm{(1}-{S}^{2})=0$, which has the analytical expression for the complex time with quadratic Stark shift

53 $$\sin \,(\frac{\omega }{{\omega }_{0}}s+\phi )=\frac{1}{{\lambda }_{0}(1+{\bar{b}}_{nlm})}[{\wp }_{z}\pm \sqrt{{\wp }_{z}^{2}+\mathrm{(1}+{\bar{b}}_{nlm})\,({\bar{b}}_{nlm}{\lambda }_{0}^{2}-\frac{1}{{n}^{2}}-{\wp }^{2})}]{\rm{.}}$$

In the absence of the Stark shifts (${\bar{a}}_{nlm}={\bar{b}}_{nlm}=0$) Eq. 53 leads to full analytical expressions, as in our previous work^31^

54 $$\sin \,(\frac{\omega }{{\omega }_{0}}s+\phi )=\frac{1}{{\lambda }_{0}}[{\wp }_{z}\pm \sqrt{{\wp }_{z}^{2}-(\frac{1}{{n}^{2}}+{\wp }^{2})}],$$

55 $${{\rm{\Omega }}}_{0}\mathcal{S}^{\prime\prime} (s,\vec{\wp })=-2{\lambda }_{0}C[{\wp }_{z}-\lambda S],$$

56 $${{\rm{\Omega }}}_{0}\mathcal{S}^{\prime} =(\frac{1}{{n}^{2}}+{\wp }^{2})-2\lambda {\wp }_{z}S+{\lambda }^{2}{S}^{2},$$

57 $$\frac{\hslash \omega }{{I}_{p}}\mathcal{S}=(\frac{1}{{n}^{2}}+{\wp }^{2}+\frac{{\lambda }^{2}}{2})\,\omega t+\lambda 2{\wp }_{z}(C-\mathrm{1)}-\frac{{\lambda }^{2}}{2}SC.$$

**Figure 2:**
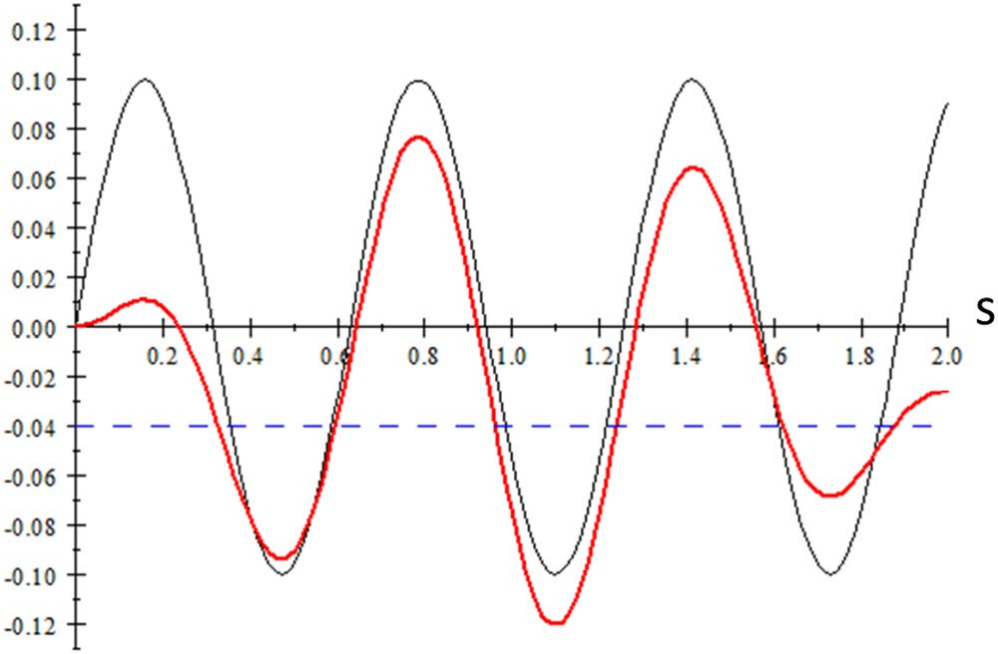
The red curve is ${{\rm{\Sigma }}}_{c}(s)={\int }_{0}^{s}g(s^{\prime} )\,\cos \,(10s^{\prime} )\,ds^{\prime}$ with pulse envelope $g(s)=s\mathrm{(2}-s)$, showing nonperiodic values of stationary points on *s* when the curve intersects the (blue) dash line which represents a constant value on the right hand side of Eq. 46. The black curve is ${\int }_{0}^{s}\,\cos \,\mathrm{(10}s^{\prime} )\,ds^{\prime}$ giving the periodic intersection points.

Comparing Eq. 54 with Eq. 46 for linear polarization, we see that effect of the pulse on the stationary point depends on solutions of ${{\rm{\Sigma }}}_{c}(s)={\int }_{0}^{s}g(s^{\prime} )\,\cos \,(s^{\prime} +\phi )\,ds^{\prime}$ instead of ${\int }_{0}^{s}\cos \,(s^{\prime} +\phi )\,ds^{\prime} =\sin \,(\frac{\omega }{{\omega }_{0}}s+\phi )$. So, there are finite number of stationary times and the times are no longer periodic, as illustrated in Fig. 2. Physically this means that the photoionization times exist only during the duration of the pulse and there is a gradient force asociated with the short pulse envelope that distorts the periodicity of the tunneling times.

## Results and Discussions

### Where we stand

Our semi-analytical method within the Keldysh framework without the saddle point approximation used to compute 3D figures of angular-dependent photoelectron energy spectra is useful, efficient and unique compared to all other existing approaches. It is in between the full TDSE and the analytical Keldysh model. The use of analytical matrix element is most unique and advantageous as it bypasses the need for three-dimensional spatial integrations, facilitating the computation of the angular-dependent photoelectron energy spectra in 3D to be more efficient and less expensive than the TDSE, and more accurate than the saddle point approximation adopted in many other existing works. To see where our work stands, let us remind the main existing works. Often, the saddle point approximation was used^59^ while going beyond the first order Born approximation^60^. Recent study of the time evolution of the photoelectron peaks used SFA without saddle point approximation^61^ but did not show angular-dependent spectra. The PPT theory^25^ is for cw laser although it was used as a semi-empirical model capable of predicting the rate of tunnel ionization for molecules^62^. Popov^63^ incorporated ultrashort laser pulses into the PPT theory using the imaginary time method (ITM) for limiting cases of tunnelling ionization and MPI but without proper atomic transition factor. In his full analytical calculations the photoionization rate was written in exponential form as generalizations of Keldysh theory at the expense of certain approximations, namely quasiclassical approach ($E\ll \tfrac{{m}_{e}^{2}{e}^{5}}{{\hslash }^{4}}$ and $\tfrac{{I}_{p}}{\hslash \omega }\gg 1$) and cannot be used to compute the angular distributions of the photoelectron spectra. The review ref. 19 of the physics of photoionization by arbitrary laser pulses shows that only for small and large Keldysh parameter *γ* it is possible to obtain analytical expressions for photoionization rate. Photoionization rate for few-cycle pulse was also obtained within adiabatic approximation^64^ by modifying the Smirnov–Chibisov formula and multiplying by an overlap integral, but introduction of the Stark shift^55^ did not yield consistent agreement with experimental results. Faisal^27^ and Reiss^28^ added higher order rescattering terms to the formula for the transition amplitude to obtain more precise results. They obtained the photoelectron spectrum (without the saddle-point technique) by expanding the final state wave function as an infinite (Fourier) series^65^. The series expansion approach is only valid for monochromatic laser fields and not valid for arbitrary pulses. Here, we do not need to evaluate infinite series and neither saddle point approximation nor adiabatic approximation are employed.

### Benchmarking

**Figure 3:**
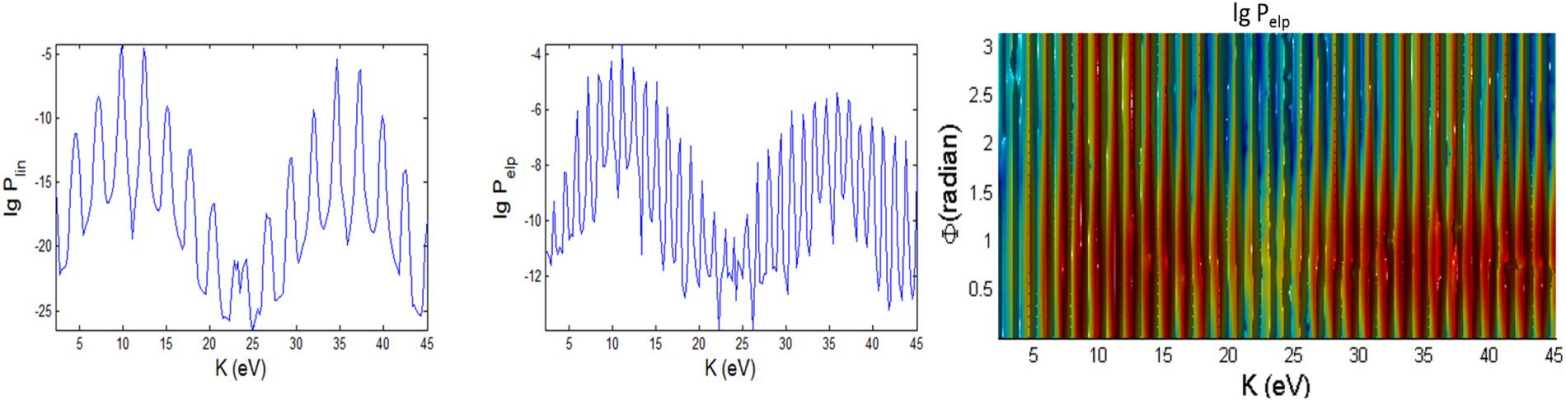
Photoionization probability versus angle Φ at Θ = *π*/2 (in x-y plane) and kinetic energy (*K*) with *t*_*p*_ = 100 fs for linear and elliptical polarized lasers for the Case A (intermediate regime) whose laser field amplitude ${ {\mathcal E} }_{0}$ and frequency *ω* are tabulated in Fig. 1. The 2D spectra correspond to the end point of Φ = *π*. Here, there is no first and second order Stark effects, ${a}_{nlm}={b}_{nlm}=0$ and the CEP phase *φ* = 0. We do not show the 3D plot for linear polarization since it has azimuthal symmetry and therefore independent of angle Φ.

**Figure 4:**
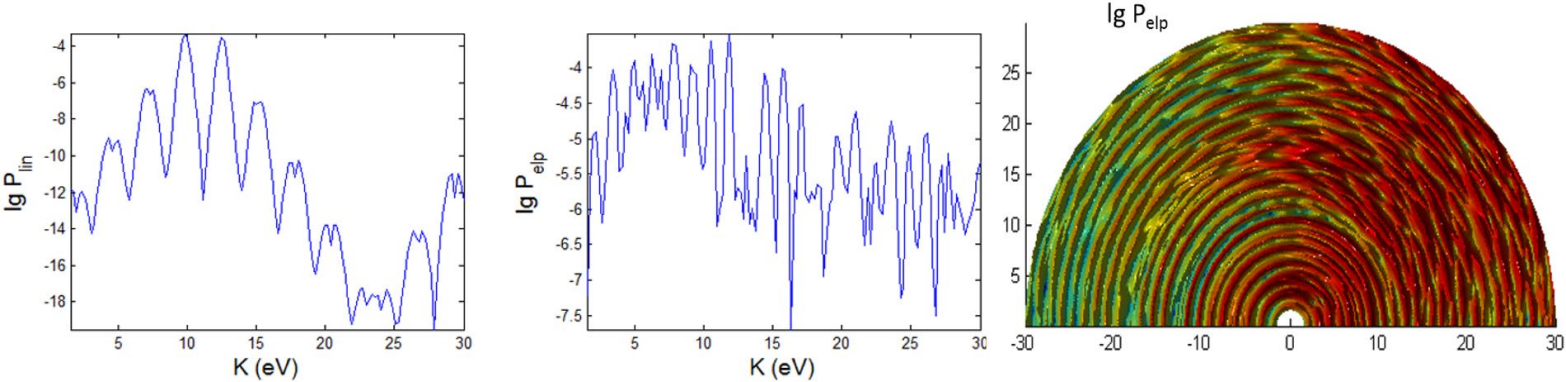
Same as Fig. 3 (versus angle Φ with *t*_*p*_ = 100 fs) but for case B.

We *emphasize* that our results are unique (*no* existing works have done this) as we analyze the effects of short pulse duration, Keldysh parameter *γ* and Stark shift on the angular distribution of photoelectron energy spectra. The 3D photoelectron angular distributions for hydrogenic system appear spectacular and may be benchmarked with the work by Martiny and Madsen^42^, probably closest to ours, who computed the photoelectron momentum distributions for few cycle pulse using the Keldysh theory without the saddle point method. However, their study focuses on how the validity of the Keldysh theory with the saddle point method depends highly on the ellipticity of the laser pulse. They found intricate details of the momentum distribution when the saddle point approximation is not used, in agreement with our results. But they did not cite earlier experiment on inert atoms that showed helicity of elliptically polarized pulses produces asymmetric photoelectron angular distributions^66^. The effects of carrier-envelope phase difference have also been studied^43^. Qualitatively similar features are found in the photoelectron momentum angular distributions of ${{\rm{H}}}_{2}^{+}$ using ab initio method by Fernandez and Madsen^67^ and of xenon atoms using quantum trajectory^68^. In addition, the results obtained from our semi-analytical approach can also be benchmarked with those 2D plots in literatures. For example, the photoelectron spectra of Figs 3, 4, 6 and 7 for longer pulse agree well with experimental and numerical results from TDSE^69, 70^. The general features of our photoelectron peaks agree with the typical ATI photoelectron spectra of helium atom for linearly and circularly polarized lasers^71^, and the ATI spectra of xenon and krypton^72^ that show regularly spaced peaks with the central energy of the group of peaks and the width of the peaks increase with the laser intensity. The ATI photoelectron spectra show no abrupt cutoff ^73^ but decrease smoothly with kinetic energy, different from HHG spectra where the cutoff at maximum kinetic energy of 3.17 *U*_*p*_ is due to electron recollision with the parent nucleus^74^, while the ATI spectra in molecules that show two cutoffs at high energies are due to scattering of the tunnelled electron from the molecular sites^75^.

**Figure 5:**
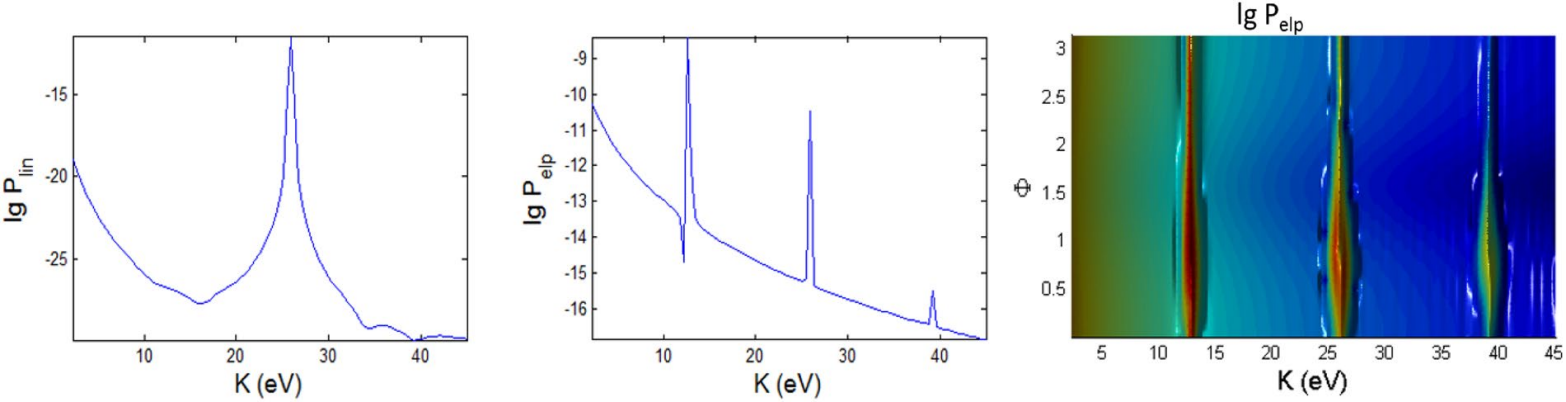
Same as Fig. 3 (versus angle Φ with *t*_*p*_ = 100 fs) but for case C.

**Figure 6:**
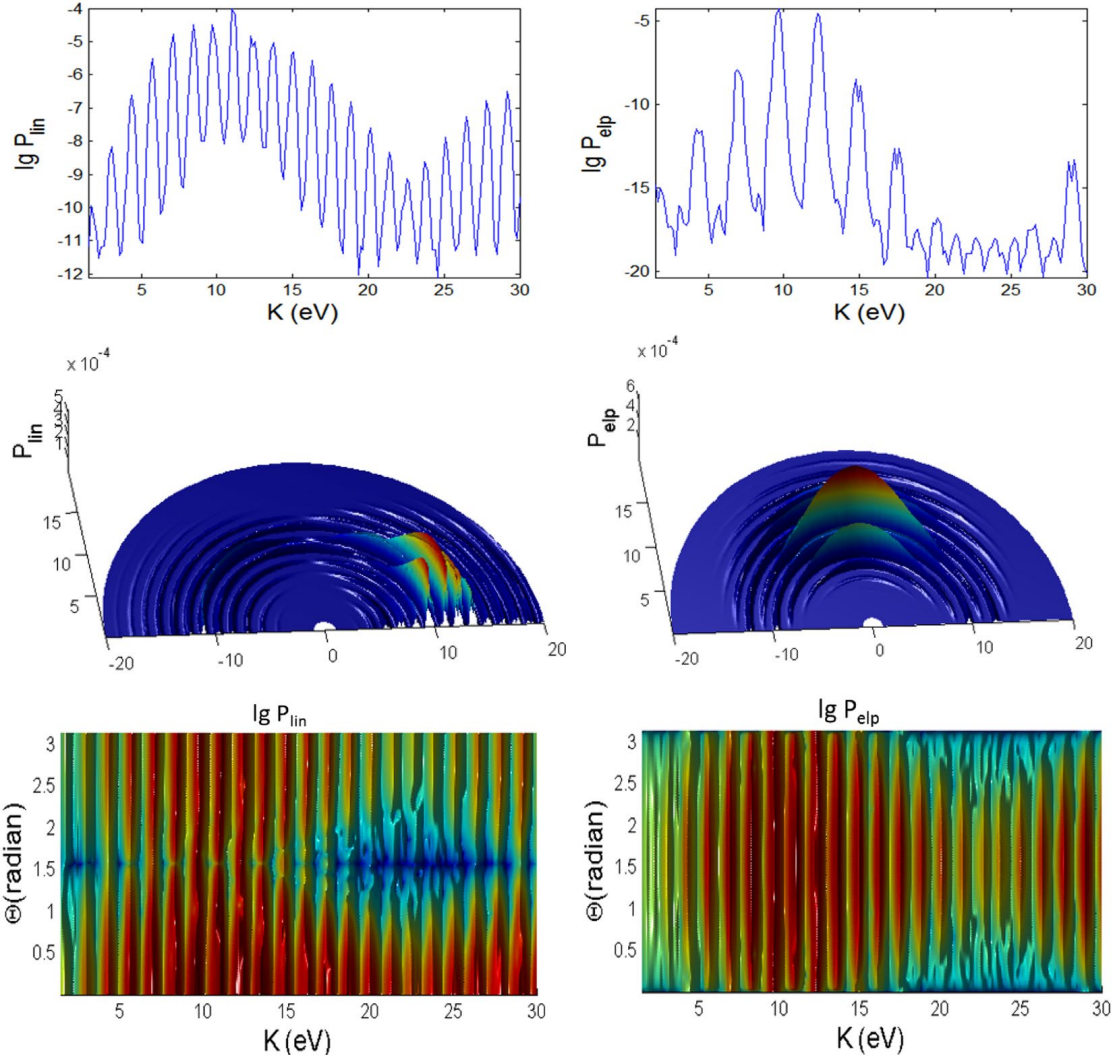
Photoionization probability with *t*_*p*_ = 100 *fs*, versus angle Θ at Φ = 0 and kinetic energy (*K*) for case A characterizing the intermediate regime. The bottom panels are *K*-Φ map with probability in log scale. The is no Stark effect and no CEP phase.

**Figure 7:**
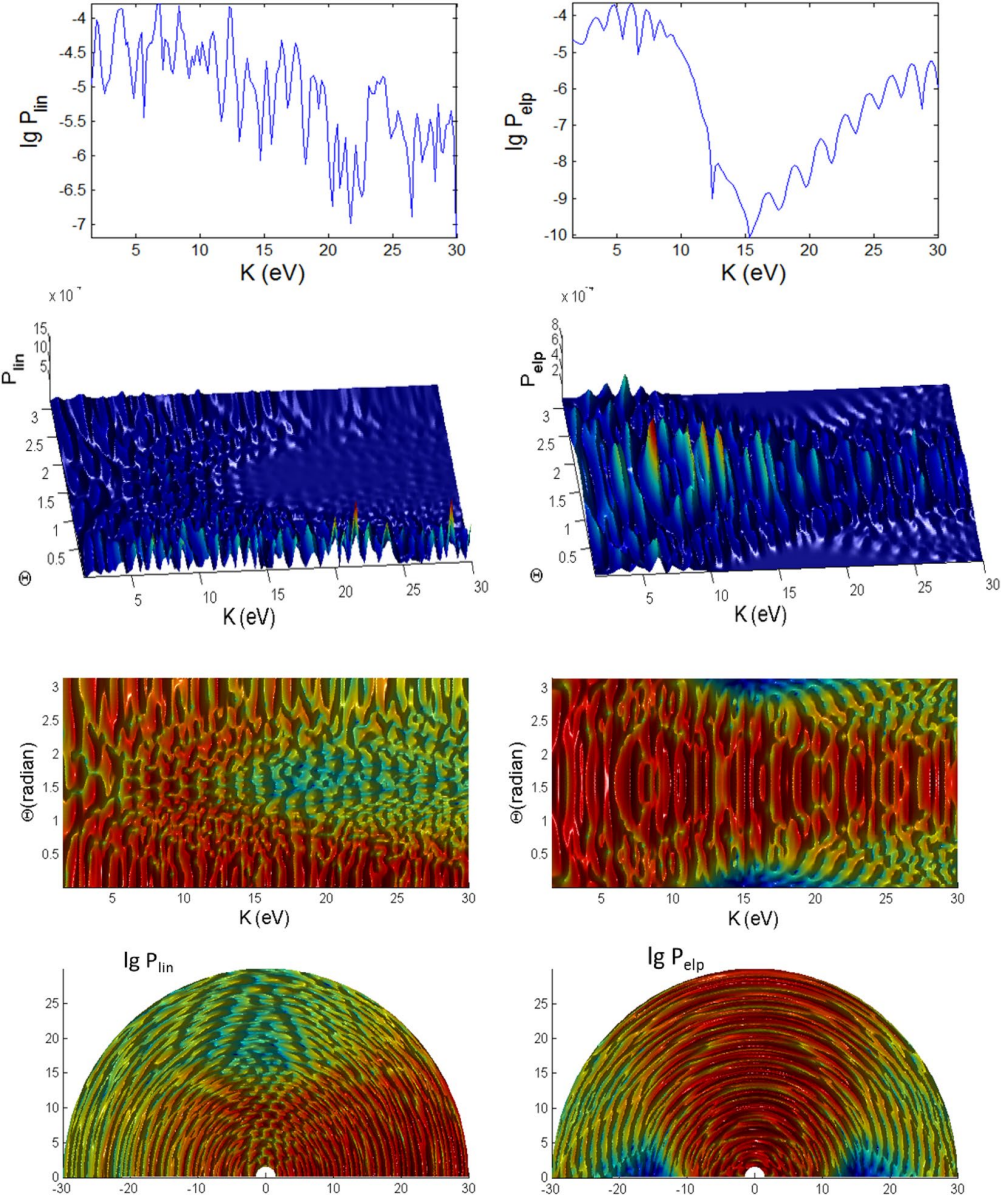
Same as Fig. 6 (photoionization probability for *t*_*p*_ = 100 *fs*, versus Θ at Φ = 0 and *K*) but for case B characterizing the ATI regime.

**Figure 8:**
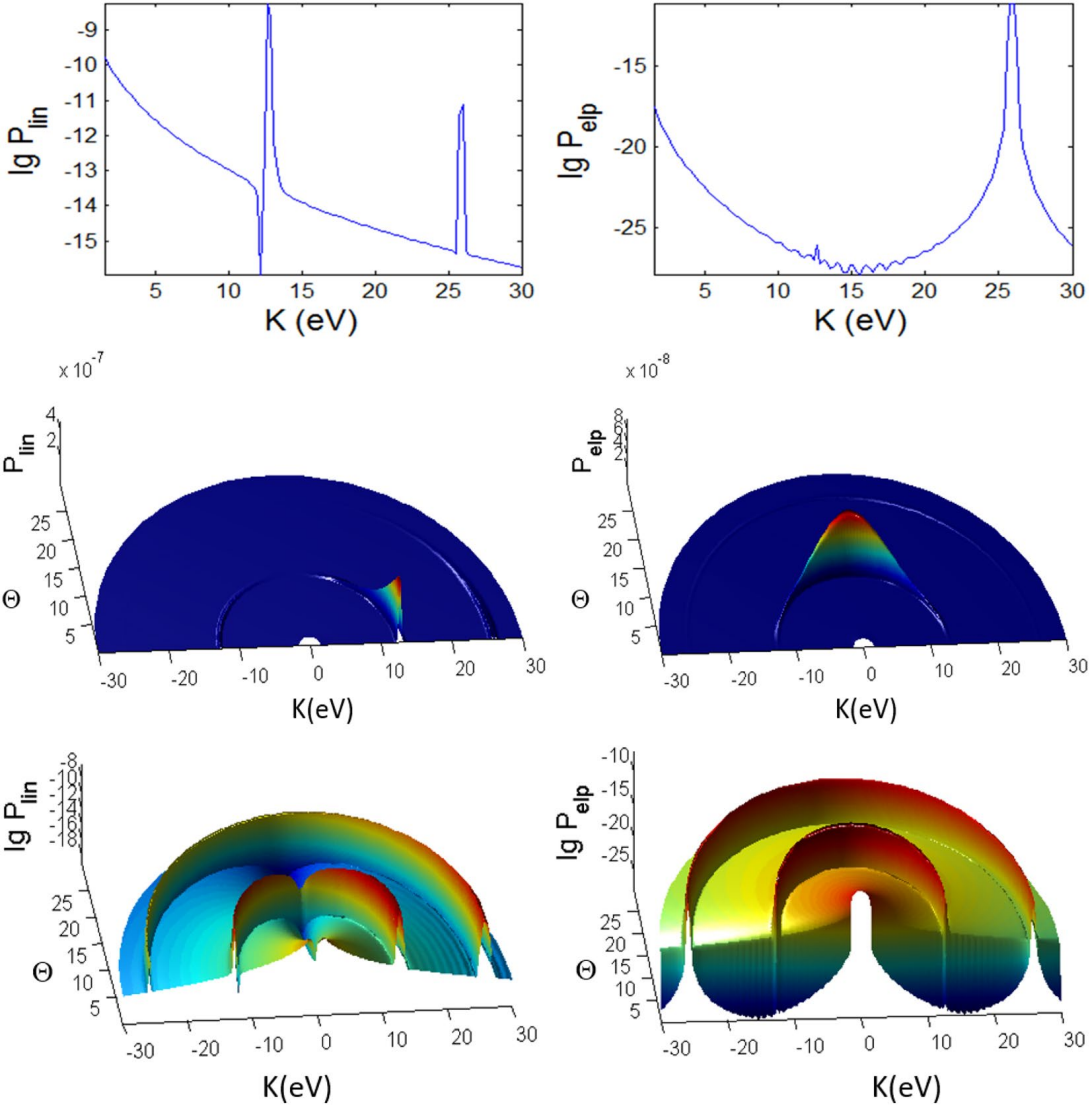
Same as Fig. 6 (photoionization probability for *t*_*p*_ = 100 *fs*, versus Θ at Φ = 0 and *K*) but for Case C characterizing the MPI regime.

In Figs 3, 4, 5, 6, 7 and 8, the photoionization probabilities at the end of the pulse, $P({\bf{p}})=\mathop{\mathrm{lim}}\limits_{s\to 2{s}_{0}}{|{c}_{b}({\bf{p}},s)|}^{2}$, for linear and elliptical polarized lasers, *P*_*lin*_ and *P*_*elp*_ (with $\alpha =\beta =\sqrt{0.5}$) are plotted versus kinetic energy *K* and observation angles ${\rm{\Phi }}$, ${\rm{\Theta }}$ for moderate pulse duration *t*_*p*_ = 100 fs. We use the initial atomic state *nlm* = 1, 0, 1. Results for hydrgenic atoms with any initial excited states can also be obtained from the theory. Based on Eq. 1 in Appendix A, the parabolic function for the pulse, the laser interaction duration is 2*s*_0_ and we have defined a normalization frequency *ω*_0_ such that ${\omega }_{0}{t}_{p}=1$, where ${t}_{p}={t}_{0}$ is defined as the pulse duration. For *n* = 1, *I*_100_ = 0.5 a.u. We consider the following three cases A, B, C, as tabulated in Fig. 1b. In the simulated figures, the first and second order Stark effects are neglected *a*_*nlm*_ = *b*_*nlm*_ = 0 with zero CEP *φ* = 0, unless stated otherwise. We show the results for *t*_*p*_ = 100 fs and for *t*_*p*_ = 10 fs (shorter pulse), corresponding to 32 and 3.2 cycle pulses, respectively.

The K-${\rm{\Phi }}$ and K-${\rm{\Theta }}$ maps of spectral distributions look qualitatively distinct for all the three regimes (A, B and C) with different electric fields and frequencies, corresponding to the intermediate, ATI and MPI regimes, respectively, and cannot be described by the classic theory^76^. The values of the probability for case B is slightly larger than case A, which implies that higher laser field amplitude increases the probability of photoionization.

#### Polarizations

**Figure 9:**
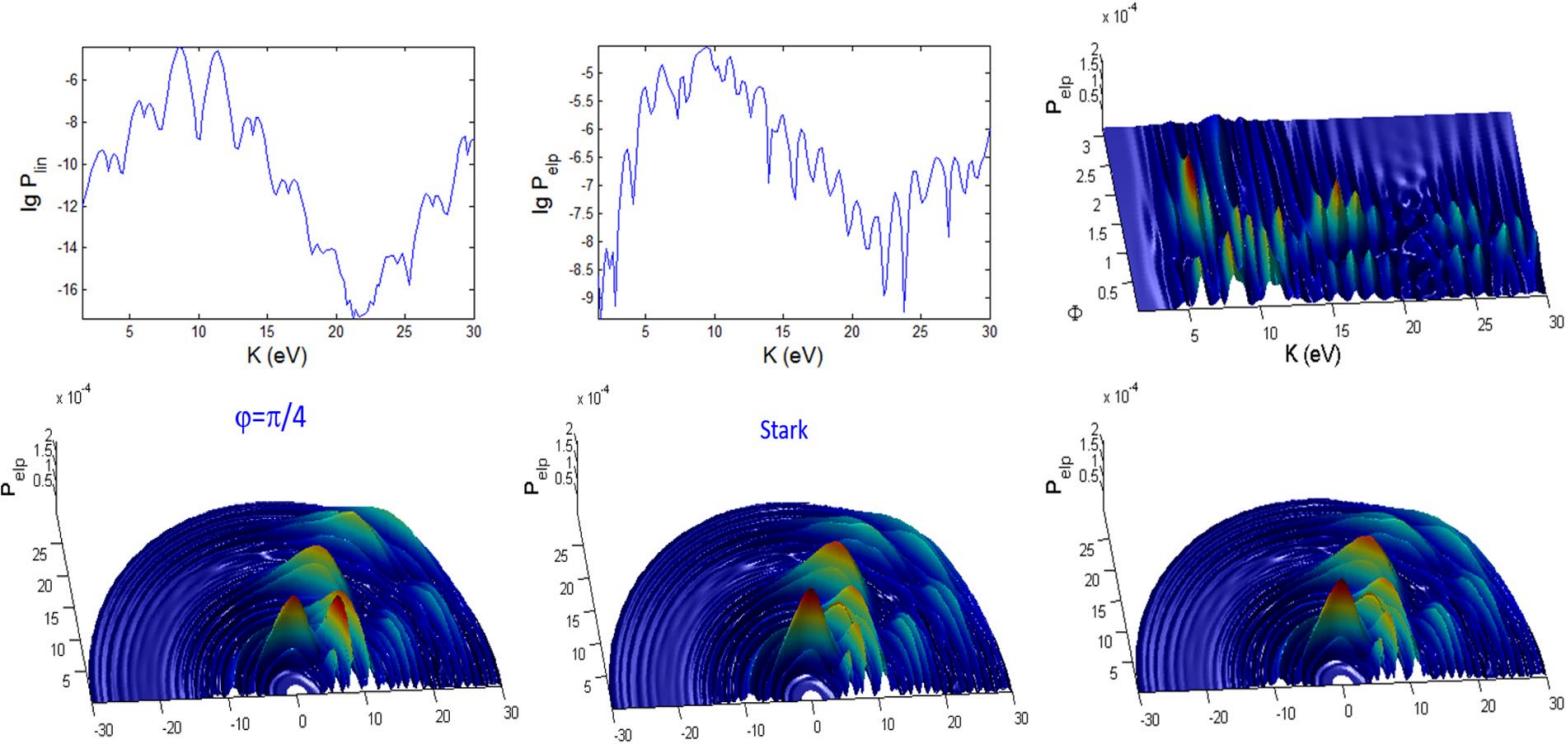
Photoionization probability with *t*_*p*_ = 10 fs versus angle Φ at Θ = *π*/2 and kinetic energy *K* for the case A (intermediate regime). There is no Stark effects and the CEP phase *φ* = 0, unless stated otherwise. Only the panels labelled with **Stark** have finite coefficient ${b}_{nlm}E{(t)}^{2}=0.8{I}_{p}\approx 10\,{\rm{eV}}$.

**Figure 10:**
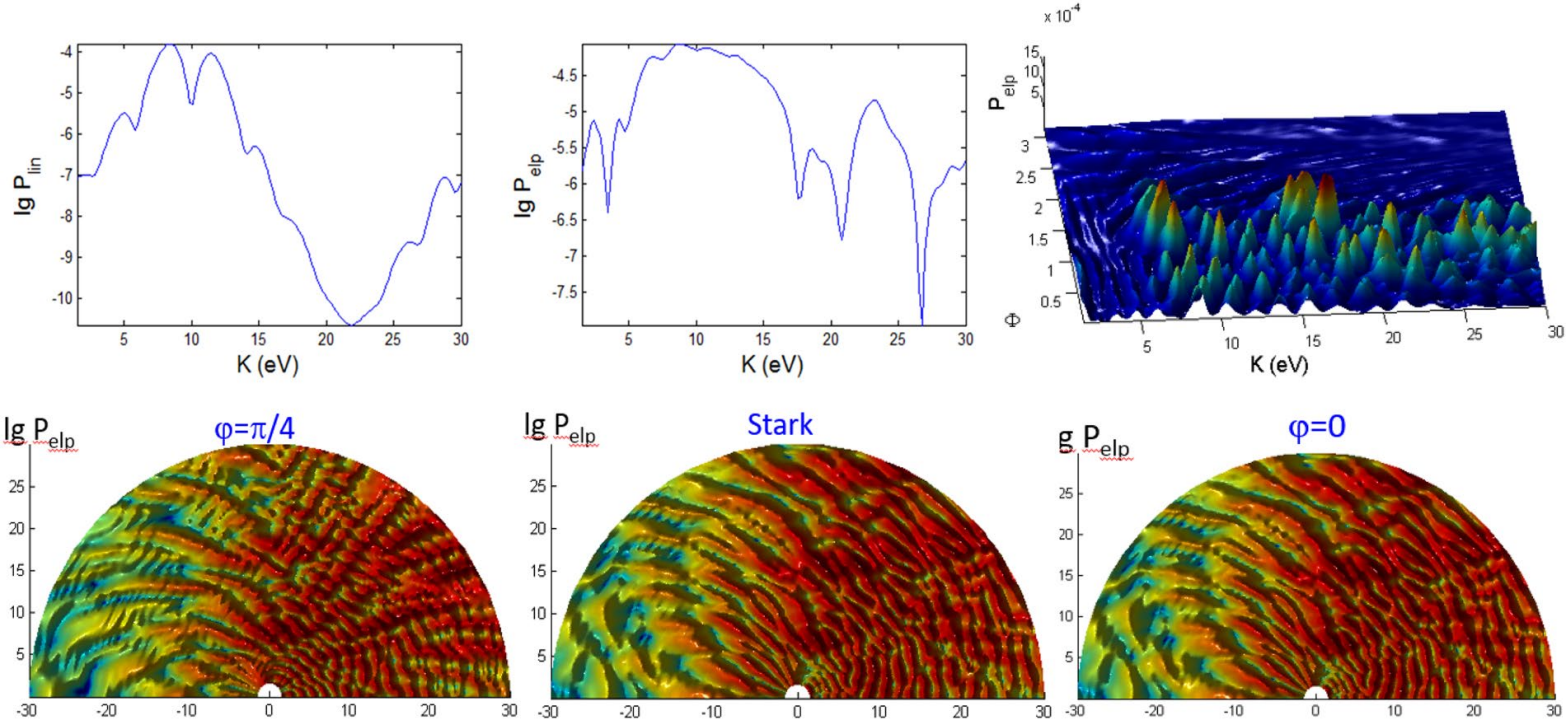
Same as Fig. 9 (versus angle Φ with *t*_*p*_ = 10 fs) but for case B.

**Figure 11:**
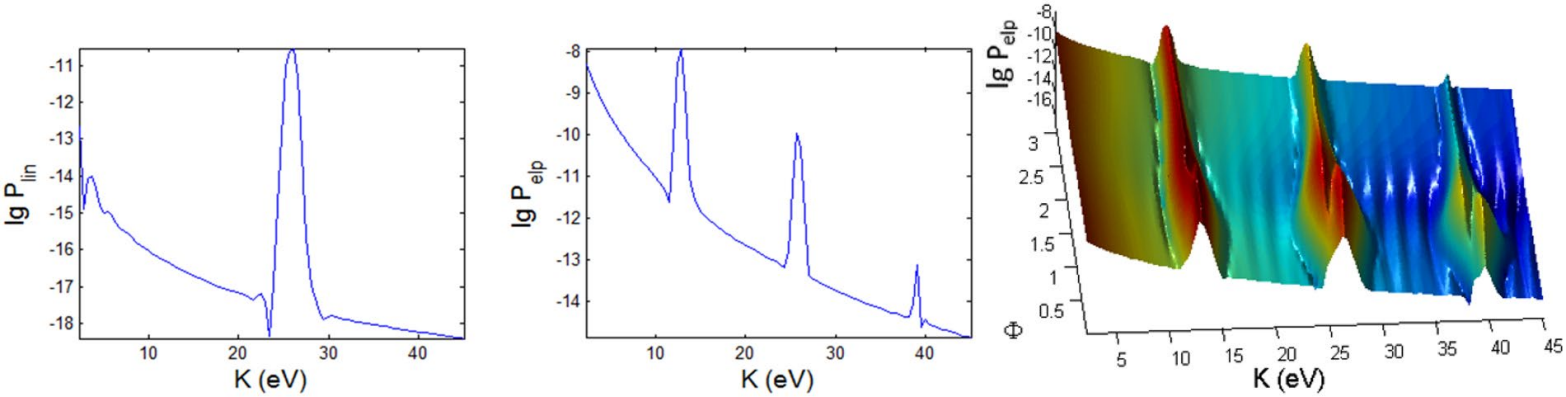
Same as Fig. 9 (versus angle Φ with *t*_*p*_ = 10 fs) but for case C.

For linearly polarized laser there is no ${\rm{\Phi }}$ dependence in the spectra, thus only the 2D plots are shown in Figs 3, 4 and 5 for longer (100 fs) pulse and in Figs 9, 10 and 11 for shorter (10 fs) pulse. The spectra for linear polarization in the intermediate cases (Figs 3 and 6), the ATI case (Figs 4 and 7) and the MPI case (Figs 5 and 8) clearly show that the peaks are regular spaced by exactly $2\hslash \omega =2.63\,{\rm{eV}}$ for ${\rm{\Theta }}$ = 90°. For other angles of ${\rm{\Theta }}$, the spacing between peaks is always $\hslash \omega$ for any polarization. However, for elliptical polarization, the peaks are always spaced by $\hslash \omega =1.36\,{\rm{eV}}$. The spectra with linear polarized laser show only even-ordered peaks at energies ${K}_{n}^{lin}(\frac{\pi }{2})=n2\hslash \omega$ at ${\rm{\Theta }}$ = 90° (perpendicular emission) as the result of destructive interference and can be confirmed by looking at the plots versus ${\rm{\Theta }}$ in Figs 6, 7 and 8. This effect was also found in ref. 77 for linear polarized laser.

#### Peak Energies

For intermediate and MPI cases, the peaks appear more regularly, and we can clearly see a dip region with the lowest probabilities at *K* ~ 24 eV, especially for linear polarization with the longer pulse of 100 fs. For the intermediate case (Fig. 3), the emission of the photoelectron is at regular energies with the peaks modulated with a period of about 24 eV.

However, the high peaks in the ATI case (Figs 4 and 7) for elliptical polarization are not regularly spaced but correlated to the angles ${\rm{\Phi }}$ and ${\rm{\Theta }}$. A closer look at the 3D plots reveals that the peaks are actually split into doublets (as in the case of molecular ion oxygen^78^) and shifted depending on the angles. Also, the high peaks are found at a broad range of energies but confined to only ${\rm{\Phi }}$ < 90°. The ATI peaks satisfy the energy conserving relation $n\hslash \omega ={I}_{p}+{U}_{p}+{p}^{2}/2m$ ^79^. The peaks for elliptical polarization are slightly smaller than linear polarization since the field is spread over the radial directions. Thus, the peak photoionization in the elliptical polarization case is slightly smaller for ATI since the tunnelling mechanism strongly depends on the transverse momenta induced by the transverse electric field.

In the MPI regime the peaks appear consistently at $n\hslash \omega =n13.16\,eV$ (Fig. 5) with the first peak missing for *Phi* = 0° and 180°. Subsequent peaks are too weak to be seen on normal/linear scales as the probability drops rapidly with energy. Thus, the log scale is useful as it clearly shows the regularly spaced subsequent peaks in the spectra.

#### Variations over Φ

The spectra for linear polarization are independent of Φ due to azimuthal symmetry (as *m* = 0 here) for any field strength ${ {\mathcal E} }_{0}$ and frequency *ω* or *γ* since the laser field oscillates along the z-axis. However the spectra with elliptical polarization for the intermediate, ATI and MPI regimes depend on the angle Φ. Figures 3, 4 and 5 show that there is *no* well-defined direction with large photoelectron emission.

In the intermediate case (Fig. 3), the spectra for elliptical polarization are less correlated to the change of angle Φ in the case of longer pulse compared to the case of shorter pulse, except for a dip (seen in log scale) that creates a double peak at around Φ ~ 0.7. The high peaks are mainly at angles 0° < Φ < 90° but only peaks at low energies appear at all angles. Only the amplitude of the peaks (and not the energy) is correlated to Φ. For the ATI case (Fig. 4), the strongest peaks are also predominantly confined within 0° < Φ < 90°. However, here, the amplitude of the peaks as well as the energy of the peaks are correlated to Φ. This can be regarded as *angular dispersion*. The angular dependence is stronger in the ATI regime (for elliptical polarization), with double peaks extending through the angle Φ and energy *K*. This multipeak effect found here for the initial ground state in the ATI case is similar to the multipeak found for the excited states by Bauer^80^.

At lower frequencies or higher field (*γ* < 1 ATI regime), $|{\lambda }_{0}\hat{a}|$ would be well above unity and greater $|\overrightarrow{\wp }|$. Thus, the normalized generalized moment ${\bf{Q}}(s)=\frac{{\rm{\Pi }}(s)}{\sqrt{2{m}_{e}{I}_{0}}}=\overrightarrow{\wp }-{\lambda }_{0}\hat{a}(s)$ would be dominated by $|{\lambda }_{0}\hat{a}|$ which causes the ${\rm{\Pi }}(s)$ to depend strongly on ${\rm{\Phi }}$, ${\rm{\Theta }}$ through the term $\exp (-\tfrac{i}{\hslash }{\rm{\Pi }}(s)\cdot {\bf{r}})$ in the transition matrix element Eq. 28. The Φ dependence is connected to the preferential photoionization between the certain sign of magnetic quantum number *m* of the bound electron and the elliptically polarized laser field^17^.

A closer look at the MPI plot (Fig. 5) reveals that the width of the peaks is modulated by the change over Φ, becoming higher and broader as Φ decreases toward Φ ~ 45°, the estimated angle where the peak splits into doublet for shorter pulse (Fig. 11). These spectra are quite identical to those found in experimental work with pulsed lasers^81^. The results for MPI also show that the shape and position of the peaks depend on the pulse duration, intensity and photon frequency^82^.

#### Variations over Θ

Figures 6, 7 and 8 show the angular dependence on Θ for linear and elliptical polarizations with longer pulses *t*_*p*_ = 100 *fs*. For linear polarization the photoelectron is mainly emitted in the direction close to Θ = 0° but there are also noticeable (but less prominent) peaks at low energies close to the backward direction 180°. For elliptical polarization (right panels of Figs 6, 7 and 8), the most probable direction is Θ ~ 90°, corresponding to the field **E** which is on the x-y plane since the photoelectron is emitted mainly along the direction of the electric field. Here, the spectra has a reflection symmetry across Θ = 90°. However, a closer look at the 3D plots for both polarizations reveals the distinctly different features in the spectra of the three cases. The spectra show the energy and angular dispersions, i.e. the heights of the photoionization probability peaks depend on *K* and Θ.

**Figure 12:**
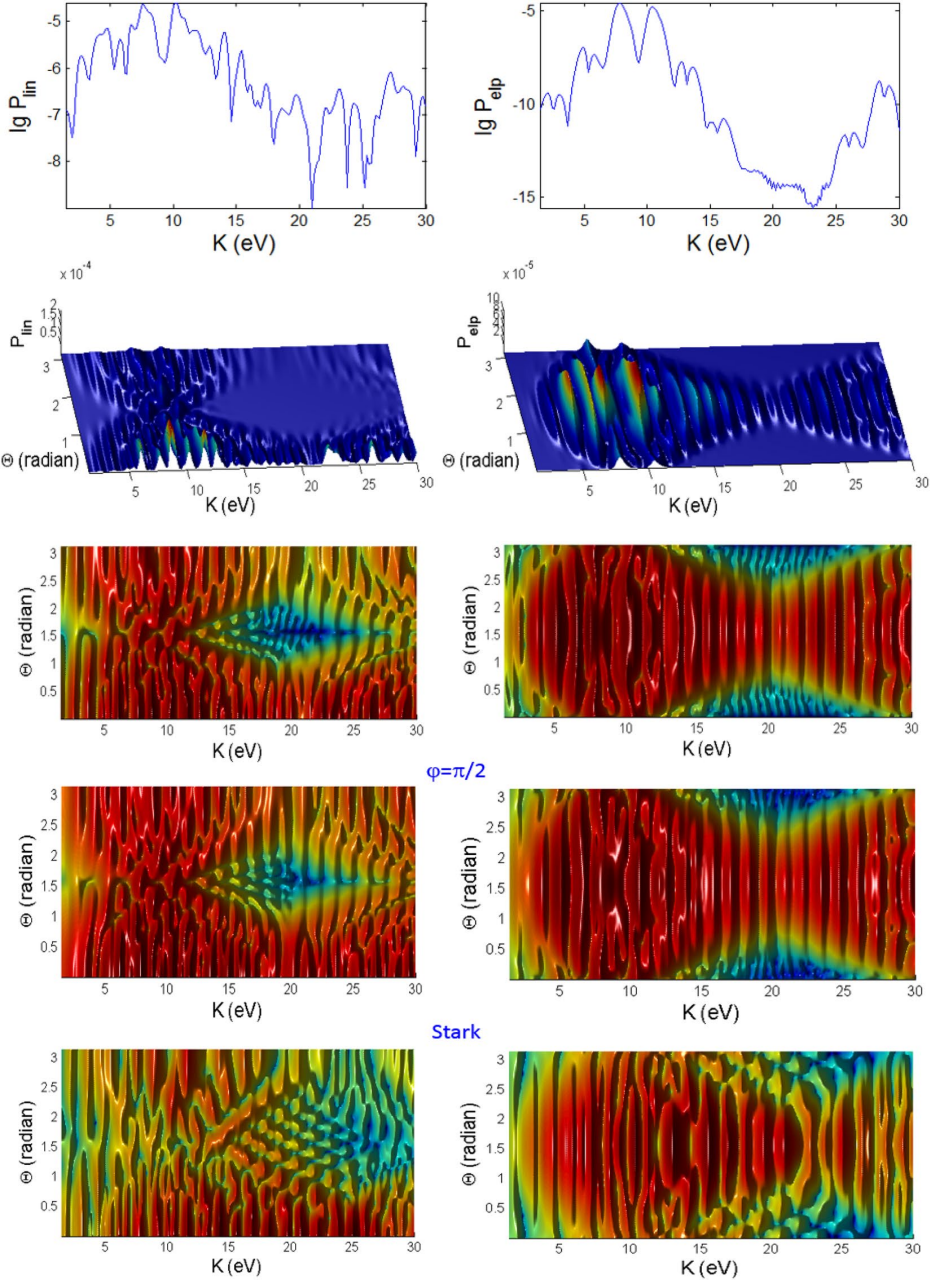
Photoionization probability for *t*_*p*_ = 10 *fs*, versus angle Θ at Φ = 0 and kinetic energy (*K*) for Case A characterizing the intermediate regime, with no Stark effect and no CEP. Only the panels labelled with **Stark** have finite coefficient ${b}_{nlm}E{(t)}^{2}=0.8{I}_{p}\approx 10\,{\rm{eV}}$. The bottom panels (reddish color) are *K*-Φ map with probability on log scale.

For the intermediate regime (Fig. 6) there is neither splitting nor angular dispersion. The energy at around *K* = 24 eV when Θ = 90° corresponds to a dip region for linear polarization spectra. For elliptical polarization this region is where the photoelectron is emitted to the narrowest range of angles centered at Θ = 90°. As the pulse shortens to 10 fs, additional features appearing in the intermediate regime (Fig. 12) are strong splitting of peaks at certain energies and angles corresponding to strong angular dispersion, especially for linear polarization. The dip region for linear polarization and the narrowest angular dispersion region for elliptical polarization have shifted down to *K* = 20 eV at Θ = 90°.

**Figure 13:**
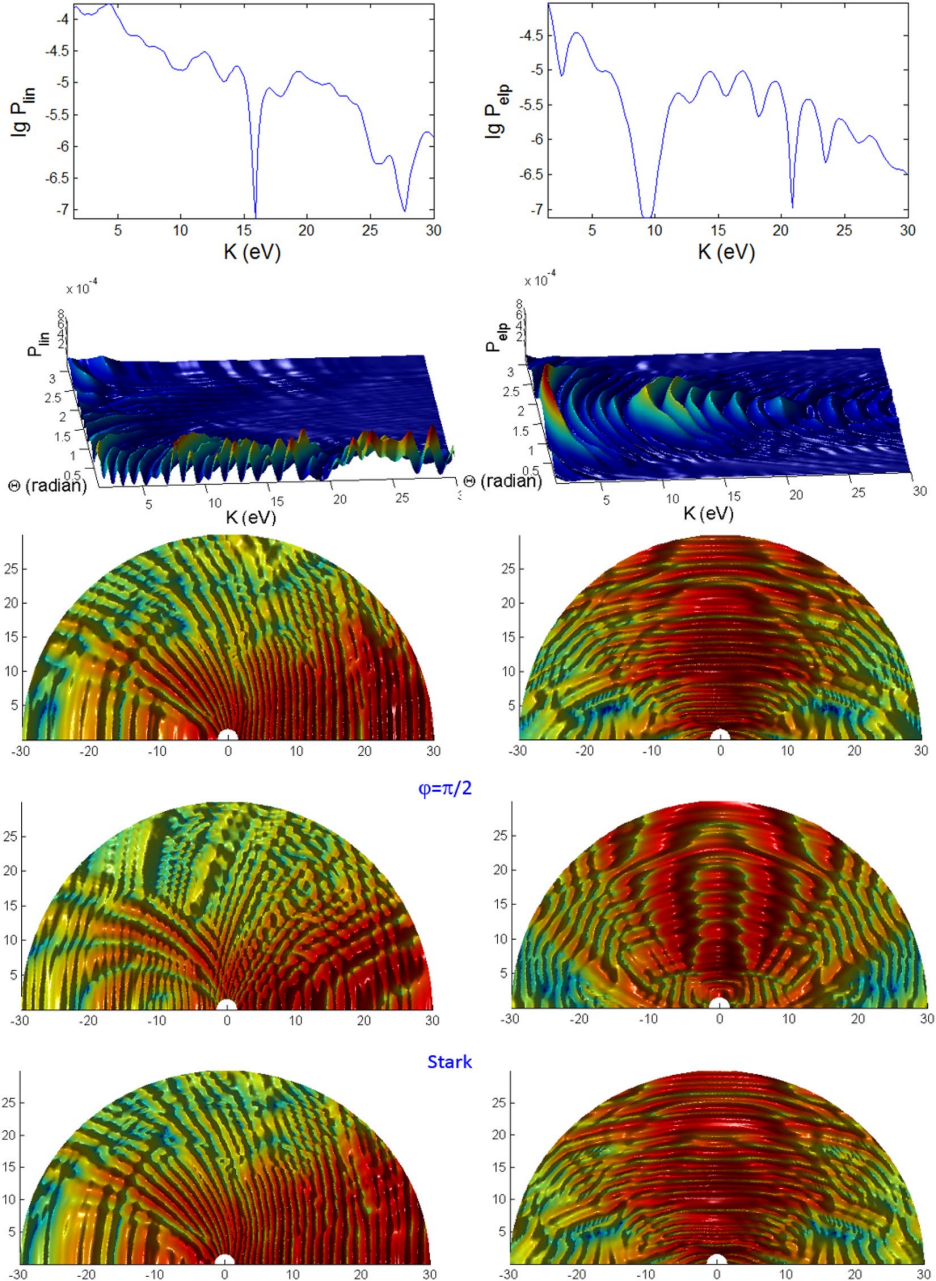
Same as Fig. 12 (photoionization probability for *t*_*p*_ = 10 fs, versus Θ at Φ = 0 and *K*) but for case B characterizing the ATI regime.

For the ATI case (Fig. 7), the emission of the photoelectron is broad, centered predominantly around Θ ~ 90° for the elliptical polarization but at around Θ ~ 0° and 180° for linear polarization. The peaks are irregular, extensively split and shited, with intricate dependence on *K* and Θ with the angular dispersion that becomes much stronger and dramatically different for shorter pulse (Fig. 13). Overall, the ATI spectra are highly correlated to the angle Θ, i.e. very sensitive to the observation direction, especially for shorter pulses. Such intricate dependence on Θ is less vivid in the case of longer pulses as the regularly spaced spectral peaks are due to excitations by photons of narrower energy range. Thus, shorter pulse leads to increased energy dispersion and more diffused photoelectron scattering.

**Figure 14:**
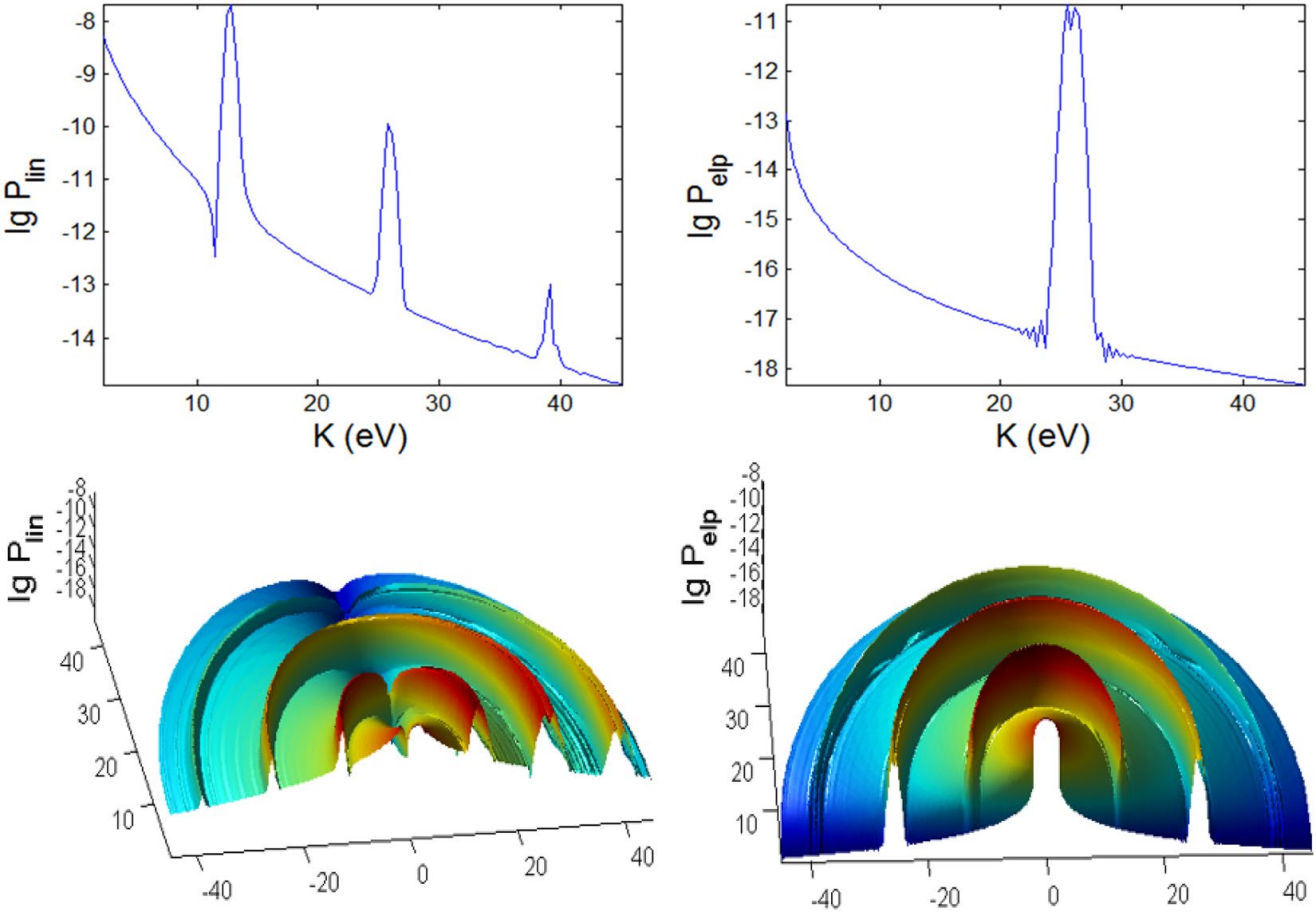
Same as Fig. 12 (photoionization probability for *t*_*p*_ = 10 *fs*, versus Θ at Φ = 0 and *K*) but for case C characterizing the MPI regime.

However, for the MPI case (Fig. 8), the angular dependence of the spectra is not so clear as the peaks are more well defined and appear only at multiples of 13.16 eV except the vanishing first peak at *K* = 13.16 eV (and odd ordered) for angles Θ = 0° and 180° in the elliptical polarization case due to destructive interference; the same for shorter pulse (Fig. 14).

#### Pulse duration effects

We found several distinct features in the photoionization of atom by ultrashort laser pulses by comparing the results for shorter pulses *t*_*p*_ = 10 fs (Figs 9, 10, 11, 12, 13 and 14) and those with longer pulses *t*_*p*_ = 100 fs (Figs 3, 4, 5, 6, 7 and 8).

The general trend of the shorter pulse *t*_*p*_ = 10 fs is that the peaks in the photoionization spectra do not only become broader^81^ but also appear to be less regular across the energy, due to splitting, shifting and overlap/crossing of peaks with angles of observation, giving rise to the stronger angular dispersion^83^. A large shift of 8 eV for $\gamma \approx 0.9$ is possible for He atom with intensity 3 × 10^14^ W/cm^2^ ^84^.

For 100 fs pulse, the peaks in the intermediate case are quite regular (Fig. 6) while for the ATI case (Fig. 7) there seem to be more peaks that are irregular, not evenly spaced and have varying widths, because of the shifts cause by the ponderomotive energy^81^ and crossings of the peaks.

The multiple frequency components within a pulse means that the photoionization mechanism is driven by photon energies within $\hslash (\omega \pm {t}_{p}^{-1})$ range. When the pulse duration becomes shorter, 10 fs (Fig. 12), the peaks in the spectra for intermediate case become irregular. In addition to splitting of the peaks, adjacent peaks merge and cross each other at particular contours, some forming “avoided crossing channels”. The asymmetry between Θ < 90° and Θ > 90° for linear polarization agrees with the findings in ref. 85 for few cycle pulses.

However, for ATI case (Fig. 13) with linear polarization, the features due to splitting have transformed to interference-like features with regular peaks appearing near Θ = 0° but strongly curved with angle Θ. This is strong angular dispersion. Similar interference and curvy peaks are found for elliptical polarization but at Θ = 90° where the spectral peaks are curved like a parabolic contour.

When the pulse duration *t*_*p*_ is shortened by 10 times to 10 fs, the maximum peaks for both polarization cases in the intermediate regime are a few times weaker (Figs 9 and 12). This shows that higher pulse energy in the case of longer pulses (for same field amplitude or peak intensity) leads to higher photoionization yield. However, the maximum of ATI peaks seem to be unaffected by the pulse duration *t*_*p*_, which shows that the pulse energy has little effect on the highest tunnelling probability (Figs 10 and 13).

The probabilities for the MPI case (in Fig. 8) do not change much except the peaks are broader for shorter pulse (Fig. 14) and for smaller Θ. For elliptical polarization the vanishing peak at Θ = 0° and 180° due to the destructive interference remains for shorter pulse. However the shorter pulse gives rise to doublets due to splitting of the peak for linear polarization at $2\hslash \omega =26.3\,{\rm{eV}}$ at Φ = 0, Θ = 0 (see Fig. 14); and also the peaks for elliptical polarization at $2\hslash \omega$ when Φ = 0, Θ = 180° (see Fig. 14) and at $2\hslash \omega$ when Φ ~ 45°, Θ = 90° (as seen in Fig. 11), as the result of destructive interference at this angle of symmetry.

#### CEP effect

We also compute the photoionization spectra for finite CEP with a (shorter) 10 fs pulse to find out if there is any qualitative effect on the overall features of the angular dependent *K*-Φ and *K*-Θ maps. For the intermediate regime (case A) the phase of *φ* = 45° (and 90°) causes small enhancement of some peaks at large energies for the *K*-Φ map (on normal scale of Fig. 9) while the finite phase of *φ* = 90° has very little effect on the intricate features of the *K*-Θ map (log scale of Fig. 12). Only the small details of the intricate patterns are affected by the phase.

However for the ATI (case B) the intricate patterns in the *K*-Φ map (when *φ* = 45° in Fig. 10) and *K*-Θ map (when *φ* = 90° in Fig. 13) are significantly different for finite phases compared to *φ* = 0. The peaks become more correlated to the angles, with traces of minima at certain angles. Thus, the tunnelling time, which is associated with the stationary time in the stationary phase analysis in Sec. VI, affects the spectra through its dependence on the absolute phase *φ*. Our results show that the qualitative change caused by *φ* is more significant when the laser field amplitude is higher. When the phase *φ* = 180° (not shown) the results are exactly the same as *φ* = 0 since it simply reverses the direction of the unit vector $\hat{n}$ and has no effect on the photoionization probability.

#### Stark effect

We introduced the first and second order Stark coefficients of ${a}_{nlm}E(t)=0$, ${b}_{nlm}E{(t)}^{2}=0.8{I}_{p}$. The second order Stark coefficient has to be sufficiently large to exhibit qualitative difference in the features of the photoionization spectra, especially for $\gamma \mathop{ > }\limits_{ \tilde {}}1$ (intermediate and MPI regimes). It is remarkable that the second order Stark has completely *no* effect on the *K*-Φ map of the intermediate and ATI cases (where $\gamma \mathop{ < }\limits_{ \tilde {}}1$ of Figs 9 and 10). This is because of the destructive interference at Θ = 90°.

However, in the intermediate case, the Stark effect displaces and redistributes the high peak structures of the *K*-Θ map (in Fig. 12) to higher energies for both linear and elliptical polarizations, with the dip (lowest probabilities) at *K* ~ 20 eV shifted to higher value *K* ~ 25 eV. This can be due to the interference between the temporal variations of the bound levels during the pulse *I*_*nlm*_(*t*) (Eq. 1) and the time dependent ponderomotive energy, *U*_*p*_(*t*), as also explained in refs 86 and 87.

For ATI regime (small frequency *ω* and large field *E*), the Stark shifts alter only some of the peaks and not the main features of the *K*-Φ map of Fig. 10 and the *K*-Θ map of Fig. 13, particularly, around Θ = 90° for elliptical polarization we see sign of modulation of the highest peaks. Thus the Stark shifts have little effect on the angular dependence of both polarizations in the ATI regime. Here, the ponderomotive energy *U*_*p*_ is larger than the intermediate case and may overwhelm the Stark energy, reducing its effect on the tunnelling mechanism.

## Conclusions

We have developed a new semi-analytical Keldysh theory for photoionization by arbitrarily short laser pulses without using the stationary phase or saddle point approximation. Using the analytical expression for the transition matrix elements obtained from the Fourier transform method, photoionization spectra can be computed more accurately and rapidly. The efficient theoretical framework without the saddle point approximation produces the 3D figures that significantly enhance our visualization of the (observational) angular dependence of the photoelectron spectra and the variations with laser pulse parameters, providing new and insightful results after analysis.

Shorter laser pulses not only give rise to broader spectral peaks that are equally spaced by $\hslash \omega$, but also crossings and splitting of peaks in the photoionization spectra. The angular dependence of the photoionization spectra for the intermediate, ATI and MPI regimes are quite distinct, with strong angular dispersion/correlation of the ATI peaks. In particular, we find the absence of the odd ordered photoionization peaks for linear polarized case at observation angle Θ = 90° due to destructive quantum interference effect, and vanishing first MPI peak with elliptical polarization at the angles Θ = 0° and 180°.

The CEP changes the detailed positions of the ATI peaks and therefore significantly alters the angular correlation of the ATI spectra, but has little effect on the intermediate case. The Stark shift has completely no effect on the azimuthal angular dependence (when Θ = 90°), has little effect on the ATI spectra but significantly shifts the photoionization spectra to higher energy for intermediate regime due to the interference between the temporal variations of the bound level and the time dependent ponderomotive energy.

Analysis of the stationary phase (compare Eqs 49 and 55) for linear polarization shows that the tunnelling times are nonperiodic due to the short pulse effect. For elliptical polarization, a larger number of stationary points appear and this is reflected in the presence of secondary peaks alluded to splitting and crossing of peaks in the photoionization energy spectra. In short, our present study has unveiled more physical insights of photoionization with ultrashort pulses, at least qualitatively, on the effects of polarization, revival of peaks by short pulse; crossing, splitting and dispersion of peaks; spectral variations with angles; CEP and Stark effects. The present semi-analytical Keldysh theory without the saddle point or stationary phase approximation would inspire further works, such as inclusion of the Coulomb corrections and relativistic effects, as well as extension to more complex quantum systems like multielectron atoms, molecular ions^88, 89^ and small molecules^90, 91^.

## Electronic supplementary material

Supplementary Information
