# Supplementary material for: Effects of ultrashort laser pulses on angular distributions of photoionization spectra
Source: Sci Rep. 2017 Jul 27;7:6739. doi: 10.1038/s41598-017-05915-8 (PMC5532372; doi:10.1038/s41598-017-05915-8)
Supplement: Supplementary file 1 — Supplementary Information [file 41598_2017_5915_MOESM1_ESM.pdf]

# Supplementary Information: Effects of ultrashort laser pulses on angular distributions of photoionization spectra

C. H. Raymond Ooi[1], W. L. Ho[1] and A. D. Bandrauk[2]

[1]*Department of Physics, University of Malaya, 50603 Kuala Lumpur, Malaysia*

[2]*Laboratoire de Chimie Théorique, Faculté des Sciences,*

*Université de Sherbrooke, Sherbrooke, Québec, Canada, J1K 2R1*

## Appendix A: Parabolic pulse

For a parabolic envelope the useful functions take the form

$$g(s) = s(2s_0 - s)/s_0^2 \tag{1}$$

where $s = \omega t$, $s_0 = \omega t_0$ and $t_0$ can be regarded is the pulse width at the base.

We need to compute the vector potential $\mathbf{A}(t)$=$-\int_{-\infty}^{t} \mathbf{E}(t)dt$ and evaluate the expression

$$\hat{a}(s) = \int_0^s g(s') \begin{pmatrix} \hat{z}\cos\left(\frac{\omega}{\omega_0}s' + \varphi\right) \\ \hat{x}\alpha\cos\left(\frac{\omega}{\omega_0}s' + \varphi\right) + \hat{y}\beta\sin\left(\frac{\omega}{\omega_0}s' + \varphi\right) \end{pmatrix} ds' = \begin{pmatrix} \hat{z}\Sigma_c \\ \hat{x}\alpha\Sigma_c + \hat{y}\beta\Sigma_s \end{pmatrix} \tag{2}$$

where we have analytical expressions

$$\Sigma_c(s) = \int g(s')\cos\left(ws' + \varphi\right)ds' = \frac{2}{s_0^2 w^3}\left((1 - \frac{1}{2}s^2w^2 + sw^2s_0)S - w(s - s_0)C\right) \tag{3}$$

$$\Sigma_s(s) = \int g(s')\sin\left(ws' + \varphi\right)ds' = -\frac{2}{s_0^2 w^3}\left((1 - \frac{1}{2}s^2w^2 + sw^2s_0)C + w(s - s_0)S\right) \tag{4}$$

with definitions $w = \frac{\omega}{\omega_0}$, $S = \sin\left(\frac{\omega}{\omega_0}s + \varphi\right)$, $C = \cos\left(\frac{\omega}{\omega_0}s + \varphi\right)$.

Similarly we need

$$\Sigma_c^{(2)} = \int_0^s g^2(s)\cos^2\left(ws' + \varphi\right)ds' = \frac{1}{s_0^4}\int s^2(2s_0 - s)^2\cos^2\left(ws + \varphi\right)ds \tag{5}$$

$$= \frac{1}{s_0^4}[\frac{1}{30}s^3\left(20s_0^2 - 15ss_0 + 3s^2\right) \tag{6}$$

$$+\frac{S_2}{8w^5}\left(3 + 2w^2\left(s_0^2 - 3\left(s - s_0\right)^2 + s^2w^2\left(s - 2s_0\right)^2\right)\right)$$

$$+\frac{1}{4}\frac{C_2}{w^4}\left(s - s_0\right)\left(2w^2s(s - 2s_0) - 3\right)]$$

$$\Sigma_s^{(2)} = \int_0^s g^2(s)\sin^2\left(ws' + \varphi\right)ds' = \frac{1}{s_0^4}\int s^2(2s_0 - s)^2\sin^2\left(ws + \varphi\right)ds \tag{7}$$

$$= \frac{1}{s_0^4}[\frac{1}{30}s^3\left(20s_0^2 - 15ss_0 + 3s^2\right) \tag{8}$$

$$-\frac{S_2}{8w^5}(3 + 2w^2\left(s_0^2 - 3\left(s - s_0\right)^2 + s^2w^2\left(s - 2s_0\right)^2\right))$$

$$-\frac{1}{4}\frac{C_2}{w^4}\left(s - s_0\right)\left(2w^2s(s - 2s_0) - 3\right)]$$

where $S_2 = \sin 2\left(\frac{\omega}{\omega_0}s + \varphi\right)$, $C_2 = \cos 2\left(\frac{\omega}{\omega_0}s + \varphi\right)$.

The dot products which we define in the text may be expressed as

$$\hat{n}\cdot\hat{a} = \begin{pmatrix} \Sigma_c \cos\left(\frac{\omega}{\omega_0}s+\varphi\right) \\ \alpha^2\Sigma_c\cos\left(\frac{\omega}{\omega_0}s+\varphi\right)+\beta^2\Sigma_s\sin\left(\frac{\omega}{\omega_0}s+\varphi\right) \end{pmatrix} \tag{9}$$

$$\hat{a}\cdot\hat{a} = \begin{pmatrix} \Sigma_c^2 \\ \alpha^2\Sigma_c^2+\beta^2\Sigma_s^2 \end{pmatrix} \tag{10}$$

$$\vec{\wp}\cdot\hat{a} = \begin{pmatrix} \wp_z\Sigma_c \\ \wp_x\alpha\Sigma_c+\wp_y\beta\Sigma_s \end{pmatrix} \tag{11}$$

The next level of integrations are

$$\int_0^s \hat{a}(s)ds = \begin{pmatrix} \hat{z}\Xi_c \\ \hat{x}\alpha\Xi_c+\hat{y}\beta\Xi_s \end{pmatrix}, \tag{12}$$

$$\int_0^s \hat{a}\cdot\hat{a}ds = \int_0^s \begin{pmatrix} \Sigma_c^2 \\ \alpha^2\Sigma_c^2+\beta^2\Sigma_s^2 \end{pmatrix} ds = \begin{pmatrix} \Xi_c^{(2)} \\ \alpha^2\Xi_c^{(2)}+\beta^2\Xi_s^{(2)} \end{pmatrix}, \tag{13}$$

where

$$\Xi_{c(s)}(s) = \int_0^s \Sigma_{c(s)}(s')ds'.$$

**Appendix B: Spherical Coordinates in Generalized Momentum Space**

The full hydrogenic wavefunction$\left(\text{dimension m}^{-3/2}\right)$ is

$$\psi_{nlm}(\mathbf{r}) = \sigma\frac{1}{(2\pi)^{1/2}}e^{im\phi}\sqrt{\frac{(2l+1)}{2}\frac{(l-|m|)!}{(l+|m|)!}}P_l^m(y)\left[(2Y)^{l+1}\sqrt{\frac{Y(n-l-1)!}{n[(n+1)!]^3}}e^{-Yr}r^lL_{n-l-1}^{2l+1}(2Yr)\right] \tag{14}$$

$$= \sigma\sqrt{\frac{(2l+1)}{4\pi}\frac{(l-|m|)!}{(l+|m|)!}}\sqrt{\left(\frac{2}{na_0}\right)^3\frac{(n-l-1)!}{2n[(n+1)!]^3}}\left[e^{-x/2}x^lL_{n-l-1}^{2l+1}(x)\right]P_l^m(y)e^{im\phi} \tag{15}$$

and its Fourier transform$\left(\text{dimension }(\text{kgms}^{-1})^{-3/2}\right)$ that was obtained analytically by Pauling and Podolsky [**?** ]

$$\bar{\psi}_s(\Pi) = \frac{1}{\sqrt{(2\pi\hbar)^3}}\int\psi_s(\mathbf{r})\exp(-\frac{i}{\hbar}\Pi(s)\cdot\mathbf{r})d^3r = \frac{1}{\sqrt{(2\pi\hbar Y)^3}}FGH \tag{16}$$

$$= \frac{1}{\sqrt{(2\pi\hbar Y)^3}}\left\{\frac{1}{(2\pi)^{1/2}}e^{\pm im\Phi'}\right\}\left\{\left(\frac{(2l+1)(l-m)!}{2(l+m)!}\right)^{\frac{1}{2}}P_l^m(\cos\Theta')\right\}\times$$

$$\left\{-(-1)^{2l+1}(-i)^l\pi 2^{2l+4}l!\left(\frac{n(n-l-1)!}{(n+l)!}\right)^{\frac{1}{2}}\frac{\zeta^l}{(1+\zeta^2)^{l+2}}C_{n-l-1}^{l+1}\left(\frac{1-\zeta^2}{1+\zeta^2}\right)\right\}$$

The generalized momentum vector and the corresponding variables in spherical coordinates are

$$= \mathbf{P}+q\frac{\mathcal{E}_0}{\omega_0}\hat{a}(s) = \begin{pmatrix} P\sin\Theta\cos\Phi + q\frac{\mathcal{E}_0}{\omega_0}\begin{pmatrix}0\\ \alpha\Sigma_c\end{pmatrix} \\ P\sin\Theta\sin\Phi + q\frac{\mathcal{E}_0}{\omega_0}\begin{pmatrix}0\\ \beta\Sigma_s\end{pmatrix} \\ P\cos\Theta + q\frac{\mathcal{E}_0}{\omega_0}\begin{pmatrix}\Sigma_c\\ 0\end{pmatrix} \end{pmatrix} = \begin{pmatrix} \Pi\sin\Theta'\cos\Phi' \\ \Pi\sin\Theta'\sin\Phi' \\ \Pi\cos\Theta' \end{pmatrix} \tag{17}$$

$$\Pi = \begin{pmatrix} \sqrt{P^2\sin^2\Theta + (P\cos\Theta + q\Sigma_c\frac{\mathcal{E}_0}{\omega_0})^2} \\ \sqrt{(P\sin\Theta\cos\Phi + q\frac{\mathcal{E}_0}{\omega_0}\alpha\Sigma_c)^2 + (P\sin\Theta\sin\Phi + q\frac{\mathcal{E}_0}{\omega_0}\beta\Sigma_s)^2 + (P\cos\Theta)^2} \end{pmatrix} \tag{18}$$

$$\tan\Phi' = \frac{P\sin\Theta\sin\Phi + q\frac{\mathcal{E}_0}{\omega_0}\begin{pmatrix}0\\ \beta\Sigma_s\end{pmatrix}}{P\sin\Theta\cos\Phi + q\frac{\mathcal{E}_0}{\omega_0}\begin{pmatrix}0\\ \alpha\Sigma_c\end{pmatrix}} = \begin{pmatrix} \tan\Phi \\ \frac{P\sin\Theta\sin\Phi + q\frac{\mathcal{E}_0}{\omega_0}\beta\Sigma_s}{P\sin\Theta\cos\Phi + q\frac{\mathcal{E}_0}{\omega_0}\alpha\Sigma_c} \end{pmatrix} \tag{19}$$

$$\tan\Theta' = \begin{pmatrix} \frac{P\sin\Theta}{\sqrt{(P\cos\Theta + q\Sigma_c\frac{\mathcal{E}_0}{\omega_0})^2}} \\ \frac{\sqrt{(P\sin\Theta\cos\Phi + q\frac{\mathcal{E}_0}{\omega_0}\alpha\Sigma_c)^2 + (P\sin\Theta\sin\Phi + q\frac{\mathcal{E}_0}{\omega_0}\beta\Sigma_s)^2}}{P\cos\Theta} \end{pmatrix} \tag{20}$$

**Appendix C: Derivatives of the Wavefunctions in Generalized Momentum Space**

The derivatives of the separated functions in $\bar{\psi}_s(\Pi)$ are

$$\frac{\partial\bar{\psi}_s}{\partial\Phi'} = im\bar{\psi}_s, \frac{\partial F}{\partial\Phi'} = imF, \tag{21}$$

$$\begin{aligned} \frac{\partial G(\Theta')}{\partial\Theta'} &= \frac{\partial}{\partial\Theta'}\left\{\left(\frac{(2l+1)(l-m)!}{2(l+m)!}\right)^{\frac{1}{2}} P_l^m(\cos\Theta')\right\} \\ &= \left(\frac{(2l+1)(l-m)!}{2(l+m)!}\right)^{\frac{1}{2}} \frac{l(\cos\theta)P_l^m(\cos\Theta') - (l+m)P_{l-1}^m(\cos\Theta')}{\sin^2\Theta'}. \end{aligned} \tag{22}$$

$$\begin{aligned} \frac{\partial H}{\partial\zeta} &= -(-i)^l \pi 2^{2l+4} l! \left(\frac{n(n-l-1)!}{(n+l)!}\right)^{\frac{1}{2}} \times \\ &\left\{\frac{8(l+1)\zeta^{l+1}}{(\zeta^2+1)^{l+4}} C_{n-l-2}^{l+2}\left(\frac{\zeta^2-1}{\zeta^2+1}\right) + C_{n-l-1}^{l+1}\left(\frac{\zeta^2-1}{\zeta^2+1}\right)\left[\frac{l\zeta^{l-1}}{(\zeta^2+1)^{(l+2)}} - 2(l+2)\frac{\zeta^{l+1}}{(\zeta^2+1)^{(l+3)}}\right]\right\}. \end{aligned} \tag{23}$$

where we have used the identity $\frac{\partial}{\partial\Theta'}P_l^m\left(\cos\Theta'\right)=\frac{l(\cos\theta)P_l^m(\cos\Theta')-(l+m)P_{l-1}^m(\cos\Theta')}{\sin^2\Theta'}$ for the associated Legendre polynomials and evaluated the derivative

$$\frac{\partial H(\Pi)}{\partial\Pi}=-\left(-i\right)^l\pi 2^{2l+4}l!\left(\frac{n\left(n-l-1\right)!}{(n+l)!}\right)^{\frac{1}{2}}\frac{\partial}{\partial\Pi}\left[\frac{\zeta^l}{\left(\zeta^2+1\right)^{l+2}}C_{n-l-1}^{l+1}\left(\frac{\zeta^2-1}{\zeta^2+1}\right)\right]. \tag{24}$$
